# School‐Based Interventions That Address Students' Unmet Material Needs: A Scoping Review

**DOI:** 10.1111/josh.70207

**Published:** 2026-08-09

**Authors:** Rebeccah Sokol, Victor Medina Del Toro, Grayson Sewall, Alexandra Swirsky, Michelle Degli Esposti, Bryan Victor, Poco Kernsmith, Justin Heinze, Judith Smith

**Affiliations:** ^1^ School of Social Work University of Michigan Ann Arbor Michigan USA; ^2^ Institute for Firearm Injury Prevention University of Michigan Ann Arbor Michigan USA; ^3^ Department of Social Policy and Intervention University of Oxford Oxford UK; ^4^ School of Social Work Wayne State University Detroit Michigan USA; ^5^ Department of Social Welfare Luskin School of Public Affairs University of California‐Los Angeles Los Angeles California USA; ^6^ Department of Health Behavior & Health Equity School of Public Health University of Michigan Ann Arbor Michigan USA; ^7^ Taubman Health Sciences Library University of Michigan Ann Arbor Michigan USA

**Keywords:** interventions, material needs, school attendance, school‐based, scoping review

## Abstract

**Background:**

Unmet material needs disrupt student learning and behavior. Some school‐based programs address these needs, but we know less about their impact.

**Methods:**

We conducted a scoping review of studies published between 2013 and 2023 in the United States that evaluated school‐based strategies to address unmet material needs.

**Findings:**

We included 55 studies and identified four intervention types: providing food, delivering healthcare, connecting families to resources, and offering transportation. Researchers have evaluated these interventions for school performance, participation, and safety. Most studies evaluated the effects of providing food on school performance (*n* = 21) and participation (*n* = 19), with mixed findings. Several studies evaluated the effects of providing healthcare on school performance (*n* = 14) and participation (*n* = 10), with overall supportive findings. There was limited evidence on connecting families to resources (*n* = 4) or offering transportation (*n* = 2). Across all interventions, only a few studies (*n* = 7) evaluated effects on school safety.

**Implications for School Health Policy, Practice, and Equity:**

School‐based healthcare can benefit school performance and participation.

**Conclusion:**

The field has focused on the effects of providing food and healthcare on school participation and performance. We can gain a more comprehensive understanding of school‐based strategies to address unmet material needs by (1) evaluating interventions beyond these two types, and (2) considering additional outcomes such as safety.

## 1 Introduction

Across childhood and adolescence, material resources—including but not limited to nutritious food, adequate clothing, reliable transportation, and stable housing—promote youth development and wellbeing [1, 2, 3]. Insufficient access to or availability of these resources creates unmet material needs among youth and their families, with direct implications for student success and school climate (e.g., absenteeism, bullying) [4, 5, 6, 7, 8]. Policies and programs addressing students' unmet material needs may improve a range of student outcomes, from academic performance to student safety. Systematically reviewing evidence can help identify which interventions schools have implemented, the outcomes that researchers have evaluated, and finally, the effects on evaluated outcomes. We undertook the present scoping review to identify the range of interventions addressing students' unmet material needs for which researchers have evaluated student learning and behavioral outcomes.

### 1.1 Prevalence and Distribution of Unmet Material Needs

The prevalence of unmet material needs among school‐aged children is high in the US [9]. During the 2022–2023 school year, over 1 million public school students experienced housing instability [10], and in 2024 over 18% of households with children experienced food insecurity [11]. Although poverty can lead to unmet material needs, many children in households with incomes above the poverty line still experience such needs [9].

Unmet material needs disproportionately burden students who already experience educational disadvantages rooted in racism and socioeconomic status [12, 13, 14, 15, 16]. In 2023, the proportion of households with food‐insecure children was over twice as high among Black and Latine households compared to White households (14% vs. 5.9%) [17]. In 2021, Latine students represented over 39% of the unhoused student population, despite representing only 28% of students [18].

### 1.2 Effects of Unmet Material Needs on Students, Schools, and Society

The effects of unmet material needs on student learning create a cycle in which racial and socioeconomic disparities in academic achievement, graduation rates, and eventually health and income, reproduce themselves. Unmet material needs can adversely affect student learning and behavior, which can have pervasive consequences across the life course and reverberating impacts for society. At the student level, unmet material needs can create barriers to regular school attendance [5, 6], limit learning and academic performance [4], become risk factors for social and behavioral problems [7, 8], and ultimately contribute to school dropout [19].

These negative consequences extend into adulthood, as adults without a high school diploma have worse labor market prospects [20], earn less [21], have more criminal justice involvement [22], experience poorer health [23, 24], and ultimately live shorter lives [23] compared to those with a high school diploma.

### 1.3 The Current Study

School‐based interventions that address students' unmet material needs may reduce a school community's level of unmet material needs while addressing foundational vulnerabilities to student learning and safety. Several reviews have described how specific types of unmet material needs—such as food insecurity [6], unreliable transportation [25], or economic hardships [26]—influence risk for specific student behavioral and learning outcomes. Yet, no review has examined the various interventions that address different material needs and their effects on student outcomes. We undertook this scoping review to define and examine the state of the science regarding school‐based interventions that address students' or their families' unmet material needs.

## 2 Methods

We registered the scoping review protocol on Open Science Framework prior to the systematic search (available at https://doi.org/10.17605/OSF.IO/T8WDZ). The JBI Manual for Evidence Synthesis informed our methodology [27]. We reported this review according to the PRISMA Extension for Scoping Reviews.

### 2.1 Search Strategy

A health sciences librarian performed our electronic search of publications via: ERIC (ProQuest), Scopus (Elsevier), Sociological Abstracts (ProQuest), Education Abstracts (EBSCOHost), Medline (OVID), and PsycInfo (EBSCOhost). We ran the search from January 1, 2013 through September 1, 2023, to capture recent studies that evaluate school‐based interventions to address unmet material needs, such as those created by the Healthy, Hunger‐Free Kids Act, the Affordable Care Act, and the Every Student Succeeds Act. We used search terms to address the three main criteria of the review: (1) school‐based; (2) intervention, policy, or program, and; (3) unmet material needs. We developed broad search terms for unmet material needs interventions after reviewing papers in different fields. Ultimately, we built the intervention terms around providing direct monetary support, connecting to services, or offering goods or services to meet a material need (e.g., clothing, books, food). The team did not include outcome terms so that the search would include various student learning and behavior outcomes. Supplemental Methods 1 provides the search strategy used in each database.

### 2.2 Inclusion and Exclusion Criteria

We included studies that evaluated the effects of school‐based interventions addressing students' unmet material needs on student learning (e.g., grades, test scores) or behavior (e.g., attendance, substance use, suspensions). We focused on student learning or behavioral outcomes given that they are commonly evaluated and both reflect and contribute to school climate. To be included, studies had to evaluate interventions within K‐12 schools in the US and be peer‐reviewed. Following these inclusion criteria, we excluded studies that were program descriptions with no evaluation, process evaluations, or review studies; did not involve interventions addressing students' or their families' unmet material needs; did not include student learning or behavioral outcomes; exclusively focused on students with disabilities and/or special education; were not school‐based; occurred exclusively in pre‐K, post‐high school, or alternative learning institutions; were not conducted in the US, or; were not peer‐reviewed. Such exclusion criteria enabled us to scope the field for general education K‐12 settings.

### 2.3 Evidence Screening and Selection

We uploaded all references from our search to Covidence systematic review software, an online platform designed to facilitate and track the screening, review, and data extraction processes [28]. For the title and abstract screening, two authors independently and blindly screened each title and abstract, and a third author resolved discrepancies. For the full‐text review, the project coordinator and lead author independently and blindly screened each full‐text and met to resolve any discrepancies. At the end of the full‐text review, we reviewed the reference lists of and articles citing the included studies to identify additional studies.

### 2.4 Extracting Data

After full‐text review, we extracted the following information: intervention characteristics (i.e., intervention description, intervention classification [universal, selective, indicated], material needs addressed); study characteristics (i.e., study aims, outcomes assessed, study design, sample size, analyses, findings), and; study setting and population (i.e., location, study years, school level, student grade and age range, student gender, and student race and ethnicity). Two data extractors independently and blindly extracted data from included studies. The data extractors and project coordinator met weekly to discuss and resolve discrepancies, consulting with the lead author as needed. The lead author then reviewed the synthesized data for all studies.

### 2.5 Quality Assessment and Synthesis

We used the Mixed Methods Appraisal Tool (MMAT) to appraise the methodological quality of included studies [29]. We used the methodology‐specific screening questions to classify studies as high, moderate, or low quality. We synthesized all included studies, regardless of their quality, and used data extraction tables to identify similarities, differences, and emerging themes.

## 3 Findings

### 3.1 Search Results

**FIGURE 1:**
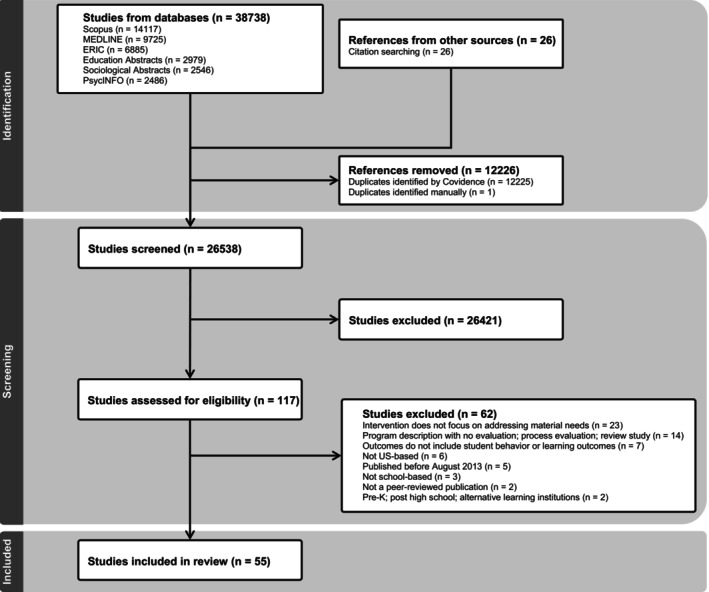
PRISMA diagram for scoping review of school‐based interventions that address students' unmet material needs, 2013–2023.

The search returned 38,764 references, of which 12,226 were duplicates. The initial title and abstract screening left 117 full texts to review, and 55 studies passed the full‐text screening (Figure 1). In the following sections, we first present the study settings and populations. Next, we describe intervention types and outcomes evaluated. We then summarize the field with an evidence map showing the intervention types and outcomes where evidence concentrates. We provide full details for included studies in the supplement for intervention characteristics (Table S1), study characteristics and findings (Table S2), study settings and population (Table S3), and quality assessments (Results S1, Tables S4–S6).

### 3.2 Study Settings and Populations

Most of the 55 included studies involved elementary schools (*n* = 44; 80.0%), 23 studies included middle schools (41.8%), and 15 included high schools (27.2%) across the US. The sampling frames for most studies were an entire school or school district and often had an equal distribution of male and female students; no studies reported the proportion of gender non‐binary or gender‐diverse students. Among the 46 studies that reported student race or ethnicity, 31 (67.4%) included schools with a majority non‐White student body.

### 3.3 Intervention Types

We categorized the 55 included studies into four distinct intervention types: providing food (*n* = 28; 50.9%), delivering healthcare (*n* = 21; 38.1%), connecting families to resources (*n* = 4; 7.2%), and offering transportation (*n* = 2; 3.6%). Each study focused on one intervention type, though some studies evaluated multiple strategies within an intervention type (e.g., evaluating universal free breakfast and breakfast after the bell in one study).

The most evaluated intervention type was providing food directly to students and/or their families at school or at home (*n* = 28; Table 1). Among these 28 studies, intervention strategies increased the availability and/or accessibility of food, including breakfast after the bell or in the classroom (BATB; *n* = 14; 50.0%), universal free meals or universal free lunches (UFM/UFL; *n* = 8; 28.6%), universal free breakfast (UFB; *n* = 6; 21.4%), the traditional School Breakfast Program (SBP; *n* = 2; 7.1%), and weekend food back‐packs (*n* = 2; 7.1%). Many schools and school districts implemented these interventions because of state or federal policies, such as the Community Eligibility Provision (CEP) or a state mandate to provide the School Breakfast Program for qualifying schools.

**TABLE 1:** Study characteristics and findings for school‐based interventions providing food directly to students or their families.

Study	Intervention	Location	School level	Student *n*	Studentgender	Student race and ethnicity	Study years	Study design	Study aims	Results
Cuadros‐Meñaca et al., 2022a	Breakfast after the bell (BATB)	Arkansas	Elementary Middle	97,527	Boys: 52.5%	Minority: 26.5%	2008–2019	Quasi‐experiment	Examine the effect of BATB on academic achievement	BATB had negative effects on math (ATT = −0.02; 95% CI: −0.04, −0.00), driven by more affluent children (ATT = −0.03; 95% CI: −0.06, −0.01). Analyses found no effects on ELA. ATT by grade indicated positive effects on math for vulnerable children with earlier BATB adoption (Grade 4 adoption: ATT = 0.10; 95% CI: 0.01, 0.19).
Abouk and Adams, 2022	Breakfast after the bell (BATB)	Texas; West Virginia; New Mexico; Nevada; Colorado	Elementary	6310	Male: 51.1%	White non‐Hispanic: 53.6% Black non‐Hispanic: 12.3% Other races: 10.1% Hispanic: 23.9%	2011–2016	Quasi‐experiment	Test for the effects of expanding school breakfast access on student achievement	Analyses did not find associations between BATB and reading, math, or science scores (*p*s > 0.05).
Cuadros‐Meñaca et al., 2023	Breakfast after the bell (BATB)	Arkansas	Elementary Middle	54,087	Not reported	Not reported	2008–2019	Quasi‐experiment	Measure the effect of BATB on the likelihood of committing an infraction and the number of infractions	Among students with prior misconduct infractions, attending a school with BATB was associated with 0.38 (SE = 0.08) fewer misconduct infractions per year. Among all students, attending a school with BATB was associated with 0.02 (SE = 0.08) fewer misconduct infractions per year. Analyses found no effects on aggressive behavior (Bs = −0.001; SEs = 0.003–0.033) or other infractions (Bs = −0.003–0.005; SEs = 0.01–0.14).
Anzman‐Frasca et al., 2015	Breakfast after the bell (BATB)	Large urban school district	Elementary	Not reported	Not reported	Black: 9.4% White: 11.4% Asian: 4.2% AIAN: 0.4% Pacific Islander: 0.5% Latino: 72.2% Filipino: 1.7%	2012–2013	Quasi‐experiment	Estimate the impact of BATB on school attendance and academic achievement	BATB was associated with greater overall school attendance rates (95.5% vs. 95.3% in the non‐BATB group; *p* < 0.01). Analyses found no group differences in standardized test performance in math (57.9% vs. 57.4% in the non‐BATB group; *p* = 0.52) or reading (44.9% vs. 44.7% in the non‐BATB group; *p* = 0.70).
Gottfried and Kirksey, 2022	Breakfast after the bell (BATB)	United States	Elementary	23,710	Male: 51.5%	Asian: 9.3% Black: 17.7% Other race: 8% Hispanic: 26.9%	2010–2012	Quasi‐experiment	Examine whether BATB is linked to better attendance	Children in schools with BATB exhibited fewer absences (*p* < 0.05) and lower likelihood of chronic absenteeism (*p* < 0.05)
Hearst et al., 2019	Breakfast after the bell (BATB)	Rural Minnesota	High	636	Female: 54% Male: 46%	White: 76% Non‐White: 24%	2013–2016	Experiment	Examine the impact of BATB on GPA	Analyses found no significant intervention effect on GPA postintervention or 1‐year follow‐up (*p*s > 0.05)
Cuadros‐Meñaca et al., 2022b	Breakfast after the bell (BATB)	Arkansas	Elementary	Not reported	Boys: 51.1%	Non‐White: 29.7%	2008–2019	Quasi‐experiment	Examine whether BATB affects math and literacy scores in third grade	BATB did not impact math or ELA standardized test scores (*p*s > 0.05).
Kirksey and Gottfried, 2021	Breakfast after the bell (BATB)	Colorado; Nevada	Elementary Middle High	1,036,384	Not reported	Asian: 3% Black: 5% Latinx: 35%	2013–2016	Quasi‐experiment	Examine the role of BATB in reducing absenteeism	Schools with BATB had chronic absence rates that were eight (SE = 0.04) to 10 (SE = 0.05) percentage points lower than schools without BATB (*p*s < 0.05). Analyses did not find an effect of BATB on reading or math (*p*s > 0.05).
Walker et al., 2021	Breakfast after the bell (BATB)	Urban district in the northeastern United States	Elementary Middle Mixed level	2906	Not reported	Not reported	2013–2016	Quasi‐experiment	Assess the effect of BATB on student absence, suspension, and tardies	School absences and school suspension rates were lower for students in the BATB program than in the Cafe program (*p*s < 0.05).
Corcoran et al., 2016	Breakfast after the bell (BATB)	New York, New York	Elementary Middle	Not reported	Male: 50.7%	Asian: 11.4% Black: 34.5% White: 13.4% Hispanic: 39.9%	2001–2012	Quasi‐experiment	Estimate the impact of BATB on students' academic performance and attendance	Analyses found no evidence of an impact of BATB on attendance or achievement (in either math or ELA exams; *p*s > 0.05).
Chandrasekhar et al., 2023	Breakfast after the bell (BATB)	Dallas Independent School District	Elementary Middle High	30,493	Male: 50.7% Female: 49.3%	Non‐Hispanic Black: 20.5% Non‐Hispanic White: 4.6% Asian: 1.2% AIAN: 0.2% NHPI: 0.1% Multiracial: 0.7% Missing: 0.03% Hispanic/Latino: 72.8%	2017–2019	Quasi‐experiment	Evaluate the impact of the BATB program on school attendance and academic performance	BATB participants were over 2.5 times more likely to attend school than non‐BATB participants (aOR = 2.55, 95% CI: 2.23–2.92; *p* < 0.001). Analyses found no significant changes in reading (*p* = 0.23) and math scores (*p* = 0.84).
Luan et al., 2022	Breakfast after the bell (BATB); Universal Free Breakfast (UFB)	Philadelphia, Pennsylvania	Elementary Middle Mixed level	1362	Male: 48.6% Female: 51.4%	Asian: 6.1% Black: 66.6% White: 7.3% Multiracial or other: 2.9% Hispanic: 17.1%	2013–2016	Experiment	Examine the effect of UFB in the classroom on attendance and standardized test performance over 2.5 years compared to UFB served before school	Analyses found no influence of BATB on attendance (B = 0.004, SE = 0.06; *p* = 0.94) or standardized reading exam scores (B = 0.02, SE = 0.06; *p* = 0.79) after 2.5 years. Students in BATB schools had lower standardized math exam scores than those in control schools after 2.5 years, although this difference was small (B = −0.20, SE = 0.07; *p* = 0.005).
Imberman and Kugler, 2014	Breakfast after the bell (BATB); Universal Free Breakfast (UFB)	Large urban school district in the southwestern United States	Elementary	6353‐ 38,425	Female: 48%	Black: 23% White: 1.5% Hispanic: 73.5%	2002–2010	Quasi‐experiment	Assess the impact of free BATB on academics and attendance relative to providing free breakfast in the cafeteria	Providing free BATB relative to the cafeteria raised math and reading achievement by 0.09 (SE = 0.046) and 0.06 (SE = 0.034) SD. Analyses did not find that BATB relative to the cafeteria influenced grades (B = −0.010, SE = 0.04) or attendance (B = −0.060, SE = 0.08).
Frisvold, 2015	School Breakfast Program (SBP)	United States	Elementary	3040–53,430	Female 49.1%–49.2%	Black: 14%–24.3% Other race/ethnicity: 3.9%–8.6% White: 60.2%–62% Hispanic 11.6%–15.4%	2003–2004	Quasi‐experiment	Investigate the impact of SBP, and a binding state mandate on school participation in an SBP program, on cognitive achievement	A binding state mandate increased math achievement by 0.9%–1.8% of the mean, reading by 0.9%–2.0% of the mean, and science by 0.8% of the mean. Exceeding the state threshold increased math achievement by 2.6 (SE = 1.24) to 7.6 (SE = 3.43) points, reading by 4.4 (SE = 1.82) to 6.6 (SE = 3.01) points, and science by 5.5 (SE = 0.39) points.
Bartfeld et al., 2019	School Breakfast Program (SBP); Universal free breakfast (UFB); Breakfast after the bell (BATB)	Wisconsin, excluding Milwaukee	Elementary	248,328–481,799	Male: 51.4% Female: 48.6%	White: 78.5% Black: 6% Asian: 4.2% Native American: 1.7% Hispanic: 9.5%	2009–2014	Quasi‐experiment	Estimate the impact of SBP on school attendance and standardized test scores	Compared with the traditional SBP, UFB was associated with a 0.24 percentage point increase in the percentage of days attended (*p* = 0.02) and a 3.5 percentage point decrease in the percentage of students with low attendance (*p* < 0.01). UFB was associated with a 0.07 SD increase in math scores (*p* < 0.01) and 0.04 SD increase in reading scores (*p* = 0.035) among higher‐income students. BATB had no associations with attendance in the full sample or subgroups. BATB was associated with lower math scores (0.05 SD, *p* = 0.045) for lower‐income boys.
Ribar and Haldeman, 2013	Universal free breakfast (UFB)	Guilford County, North Carolina	Elementary	8078	Female: 48.9%	Black: 65.9% Hispanic: 17.1%	2007–2009	Quasi‐experiment	Investigate student outcomes (attendance rates and standardized test scores) associated with changes in the availability of UFB	Analyses did not find an association between UFB (versus eligibility‐based breakfast) and student attendance. At the student level, UFB was associated with a half percent decrease in attendance (SE = 0.001; *p* < 0.01). Analyses found no evidence that UFB is associated with reading or math test results or with science raw scores. UFB was associated with science test proficiency among economically disadvantaged children (B = 0.08; SE = 0.03).
Bullock et al., 2022	Universal free breakfast (UFB)	Large urban school district in the southeastern United States	Elementary Middle High Mixed level	Not reported	Not reported	African American: 37%, White: 25% Asian American: 7% Multiracial: 3% NHPI: < 1% Hispanic: 28%	2006–2015	Quasi‐experiment	Examine whether a UFB policy was associated with changes in school attendance	Analyses did not find increases in SBP participation to be associated with significant changes in attendance (0.003 SE = 0.01, *p* = 0.9).
Leos‐Urbel et al., 2013	Universal free breakfast (UFB)	New York, New York	Elementary Middle	723,843	Not reported	Black: 32.3% Asian: 14.2% White: 17.8% Hispanic: 35.7%	2002–2004	Quasi‐experiment	Examine the impact of the implementation of UFB on attendance and academic achievement	Analyses identified no change in attendance rates (B = 0.11–0.14, SE = 0.09–0.10), reading scores (B = 0.01, SE = 0.01), or math scores (B = −0.01–0.02, SE = 0.0) after adopting UFB.
Williams, 2020	Universal free lunch (UFL)	Tennessee	Elementary	Not reported	Not reported	Not reported	2013–2014	Quasi‐experiment	Examine the impact of policy change of UFL on third grade normal curve equivalent reading scores	Analyses found no differences in third grade reading scores after the policy change to 100% UFL (*p* > 0.05).
Gordanier et al., 2020	Universal free lunch (UFL) through CEP	South Carolina	Elementary Middle	332,761	Female: 48.8%	White: 53.5% Black: 35.4% Asian and Pacific Islander: 2.1% Native Indian: 0.6% Hispanic: 8.4%	2014–2016	Quasi‐experiment	Investigate the effect of UFL, through CEP, on academic outcomes of elementary and middle school students	For elementary school students, CEP led to a 0.061 (SE = 0.014) standard deviation increase in math test scores and 0.213 (SE = 0.111) fewer absences; analyses found no effect of CEP on reading test scores for elementary students. Analyses did not find any effects of CEP on math test scores, reading test scores, or absences among middle school students.
Schwartz and Rothbart, 2020	Universal free meals (UFM)	New York, New York	Middle	645,204	Female: 50.3%	White: 15% Asian: 17.4% Black: 28.1% Hispanic: 39.5%	2013	Quasi‐experiment	Estimate the impact of UFM on student outcomes, including performance on test scores and attendance	UFM increased math and ELA scores for middle school students by 0.036 (SE = 0.014) and 0.030 (SE = 0.011) standard deviations, respectively. Analyses found no effects on attendance.
Taylor et al., 2020	Universal free meals (UFM)	Vermont	Elementary Middle High	Not reported	Not reported	Not reported	2016–2017	Cross‐sectional	Analyze the impacts of UFM on student academics and behavior	Eighty‐three percent of school staff agreed or strongly agreed that UFM make students more ready to learn; 64.4% agreed that students' academic performance had improved with universal meals. Most respondents (59.1%) agreed that student behavior improved. Most school nurse participants disagreed that student issues had declined (55%).
Andreyeva and Sun, 2021	Universal free meals (UFM) through CEP	41 states; urban, suburban, and rural areas	Elementary	17,800	Male: 51.3% Female: 48.7%	Non‐Hispanic White: 47.6% Non‐Hispanic Black: 11.3% Asian: 8.8% Other race: 5.8% Hispanic: 26.4%	2010–2016	Quasi‐experiment	Assess the impacts of CEP on attendance and academic achievement among low‐income communities	UFM improved daily school attendance by 0.24 percentage points (*p* < 0.01). Models for academic achievement outcomes did not pass the parallel trend assumption in the full analytic sample. Among Hispanic children, there was a CEP‐attributable increase in reading scores (0.06 SD, *p* < 0.1).
Ruffini, 2022	Universal free meals (UFM) through CEP	United States	Elementary Middle	Not reported	Not reported	Black: 22.4% Hispanic: 16.7%	2006–2018	Quasi‐experiment	Evaluate the effect of CEP on academic performance	Analyses did not find that CEP improved reading or math performance overall. Among exposed districts with the largest gains in access to free meals, CEP improved overall math performance by 0.02 SD (SE = 0.009).
Gordon and Ruffini, 2021	Universal free meals (UFM) through CEP	United States	Elementary Middle High	8,594,838–22,423,824	Not reported	Black: 12.3%–13.9% White: 46.5%–60.4% Hispanic: 18.6%–20.5%	2012–2017	Quasi‐experiment	Examine whether schoolwide free meals affect disciplinary outcomes, focusing on the use of suspensions	Schoolwide free meals reduced suspensions by approximately one percentage point (SE = 0.46; *p* < 0.05) for white male elementary students; analyses did not find associations overall for elementary students, middle/high school students, or for other subgroups.
Bartfeld et al., 2020	Universal free meals (UFM) through CEP	Wisconsin, excluding Milwaukee	Elementary	Not reported	Not reported	Non‐white: 48.9%	2013–2016	Quasi‐experiment	Estimate the association between CEP and school attendance among elementary school students	Implementing the CEP had no association with attendance in the initial year. The second year of CEP was associated with a 3.5 percentage point reduction in the percentage of students with low attendance (*p* = 0.045), driven by economically disadvantaged students.
Kurtz et al., 2020	Weekend food back‐pack (BackPack)	12 counties in northwestern North Carolina	Elementary	32,690–36,986	Female: 49.2%	White: 27.6%	2007–2013	Quasi‐experiment	Examine the effects of BackPack on end of grade test scores and absenteeism	Having a BackPack program increased reading test scores by 0.093 (SE = 0.039) standard deviations (*p* < 0.05). Analyses found no effects of the BackPack program on math test scores (B = 0.07, SE = 0.06; *p* > 0.05) or absences (B = 0.146, SE = 0.152; *p* > 0.05).
Fiese et al., 2020	Weekend food back‐pack (BackPack)	East central Illinois	Elementary	444	Boys: 51%	African American: 31.7% Caucasian: 54.4% Multiracial: 6.6% Latino: 6.6%	2011–2012	Cross‐sectional	Determine the potential of the Backpack program to affect school attendance	There were fewer absences on Fridays for students who participated in the BackPack program compared to Mondays‐Thursdays; this was not true for non‐recipients.

Abbreviations: AIAN, American Indian and Alaska Native; ATT, average treatment effect on the treated; BATB, breakfast after the bell; CEP, Community Eligibility Provision; CI, confidence interval; ELA, English Language Arts; GPA, grade point average; Mod., moderate; NHPI, Native Hawaiian or Pacific Islander; SBP, School Breakfast Program; SD, standard deviation; SE, standard error; UFB, universal free breakfast; UFL, universal free lunch; UFM, universal free meals.

The next most evaluated intervention type was delivering healthcare (*n* = 21; Table 2). These interventions directly provided medical care at school at little or no cost to students or their families. Among these 21 studies, intervention strategies included school‐based health centers (*n* = 6; 28.6%), vision services (*n* = 5; 23.8%), school nurses (*n* = 5; 23.8%), influenza vaccine clinics (*n* = 4; 19.0%), and mobile dentists (*n* = 1; 4.8%).

Four studies involved interventions that focused on connecting families to existing school or community resources (Table 3). These interventions focused on supporting multiple, diverse material needs among students and their families and included strategies such as referrals to food pantries, direct cash, and transportation support. These interventions included interprofessional teams and partnerships between the school and community agencies.

Two studies evaluated interventions focused solely on transportation, one examining school buses and one evaluating free public transportation for students (Table 3).

**TABLE 2:** Study characteristics and findings for school‐based interventions to deliver healthcare to students.

Study	Intervention	Location	School level	Student *n*	Student gender	Studentrace and ethnicity	Study years	Study design	Study aims	Results
Gicquelais et al., 2016	Influenza vaccine clinic	Arkansas	Elementary Middle High Mixed level	Not reported	Not reported	Not reported	2012–2013	Cross‐sectional	Quantify the association between student vaccination levels and student absenteeism during influenza season	A one‐percent increase in student vaccination rates was associated with 0.027% (adjusted; unadjusted: 0.031%) fewer days absent (*p* < 0.01).
Pannaraj et al., 2014	Influenza vaccine clinic	Los Angeles County, California	Elementary	4455	Male: 52.1%	Asian: 26.7% White: 2.0% Black: 0.6% Other: 4.3% Hispanic: 66.3%	2010–2011	Quasi‐experiment	Evaluate the impact of a school vaccine clinic on absenteeism	All‐cause absentee rates among students were higher in comparison schools compared with intervention schools (4.2 vs. 3.9 days per 100 school days; *p* = 0.02). Fewer children in comparison schools had zero days of absenteeism compared with those in intervention schools (549 [26.3%] vs. 793 [33.5%]; *p* < 0.01)
Benjamin‐Chung et al., 2020	Influenza vaccine clinic	Oakland Unified School District, California	Elementary	Not reported	Not reported	White: 43.8% Black or African American: 22.4% Asian: 17.2% Other Race: 8.6% NHPI: 0.6% Multiracial: 6% Hispanic or Latino: 28.8%	2011–2018	Quasi‐experiment	Assess whether the school vaccine clinic was associated with reduced school absences	In 2016–2017 and 2017–2018, the illness‐specific absence rate per 100 days was lower in the intervention district compared to the comparison district (2016–2017 DID: −0.63 [95% CI: −1.14, −0.13], *p* = 0.014; 2017–2018 DID: −0.80 [95% CI: −1.28, −0.31], *p* = 0.001). For all‐cause absences, the DID estimates during influenza season were not statistically significant in any years of the program.
Plaspohl et al., 2014	Influenza vaccine clinic	Effingham County, Georgia	Elementary	4674–5201	Male: 52% Female: 48%	White non‐Hispanic: 77% Black: 14% Multiracial: 4% Asian/Pacific Islander: 1% AIAN/Unknown: < 1% Hispanic: 4%	2007–2012	Quasi‐experiment	Assess the associations between a school vaccine program and student absences for elementary school students	Students in the 2 years pre‐program were absent for 0.36 fewer days compared to students in the two program years (*p* < 0.0001; 95% CI = 0.28, 0.44). Vaccinated students in the two program years were absent for 0.64 fewer days as compared to unvaccinated students in the two program years (*p* < 0.0001, 95% CI = 0.51, 0.78).
Ruff et al., 2023	Mobile dentist, CariedAway	New York, New York	Unknown	Not reported	Not reported	Not reported	2016–2021	Experiment	Examine the effect of school‐based dental programs on student attendance	Schools recorded ˜944 fewer absences during treatment compared to nontreatment years (95% CI = –1739 to −149). Analyses found no difference in absences between treatment and comparison schools (*p* > 0.05).
Darnell et al., 2019	School nurse	Kentucky	High	Not reported	Not reported	Not reported	2013–2014	Cross‐sectional	Examine the relationship between school nurse presence and graduation rates, absenteeism, and ACT scores	Schools with a full‐time nurse had a lower absentee rate (6.3% vs. 6.98%; B = −0.76, *p* = 0.01), higher student graduation rate (83.1% vs. 76.9%; B = 5.67, *p* < 0.001), and higher ACT scores (18.9 vs. 18.5 ACT; B = 0.51, *p* = 0.04) than schools with no nurse.
Rodriguez et al., 2013	School nurse	San Jose Unified School District, California	Elementary Middle	583–6081	Not reported	Hispanic/Latino: 77.4%	2006–2009	Quasi‐experiment	Examine the change in school absenteeism associated with full‐time nurses	Students in demonstration schools had fewer absences due to illness (3.03 days) compared to students in comparison schools (3.51 days; *p* < 0.05).
Leach et al., 2023	School nurse	Jefferson County, Kentucky	Elementary	16,025	Female: 48.5% Male: 51.5%	Black: 45.5% Other race/ethnicity: 9.4% White: 30% Hispanic/Latinx: 15.5%	2017–2019	Quasi‐experiment	Evaluate the effects of an existing LPN‐based school nurse program on attendance and chronic absenteeism	Analyses found no associations between being in schools with a nurse and chronic absenteeism or attendance (*p*s > 0.05).
Best et al., 2021	School nurse	All school districts in North Carolina	Elementary Middle High	1.43 million	Not reported	Not reported	2011–2016	Descriptive, longitudinal	Examine the impact of student‐to‐nurse ratios on education outcomes (i.e., absences, grades) of students receiving services for asthma and diabetes	Lower student‐to‐school nurse ratios were associated with fewer absences and improved grades (*p* < 0.05).
Lim et al., 2023	School‐based health center (SBHC)	Southern California	Unknown	244,076	Female: 56.6% Male: 43.4%	Asian: 5.9% Black: 6.0% White: 10.5% Multiracial: 0.2% Unknown: 0.7% Latine: 76.6%	2015–2020	Quasi‐experiment	Examine whether school attendance improves after students receive care at an SBHC	Visiting an SBHC was associated with a 0.65 percentage‐point increase in school days attended per month (*p* < 0.01). An SBHC mental health visit was associated with a 2.33 percentage‐point increase in school days attended per month (*p* < 0.001).
Westbrook et al., 2020	School‐based health center (SBHC)	Colorado	High	Not reported	Women: 48.9%	Minority: 22.8%	2000–2018	Quasi‐experiment	Examine the association between SBHC opening and high school graduation rates	Opening an SBHC during the study period was associated with a 4.1 percentage point increase in the overall graduation rate (*p* = 0.08).
Runton and Hudak, 2016	School‐based health center (SBHC)	Virginia; North Carolina	Middle High	750–1169	Female: 49.8%–54.9% Male: 45.1%–50.2%	White: 25.6%–25.9% Black: 38.6%–41.7% Other/multiple: 11.2%–11.7% Hispanic/Latino: 21%–24.3%	2007–2011	Descriptive, longitudinal	Investigate which health risk behaviors were affected by a new SBHC	Analyses did not find associations between SBHCs and the likelihood of a student carrying a weapon, being in a physical fight, or smoking a cigarette (*p*s > 0.05).
Strolin‐Goltzman et al., 2014	School‐based health center (SBHC)	Large, northeastern urban metropolis	Elementary Middle High	793	Female: 48.5% Male: 51.3%	African: 2.5% Black or African American: 19.6% Asian/Pacific Islander: 5.2% Non‐Hispanic White: 6.6% Native American: 1.1% Multiracial: 4.6% Hispanic: 60.2%	Not report‐ed	Cross‐sectional	Evaluated associations between SBHC use and academic performance	SBHC users in the middle and high schools had GPAs that were 2.5 points higher than those for nonusers (*p* < 0.05). 90% of middle and high school SBHC users advanced to the next grade, compared to 83% of non‐users (*p* < 0.05). SBHC users were late on an average of 9.5 days, whereas nonusers averaged five tardy days (*p* < 0.05). There was no difference in attendance between users and nonusers (*p* > 0.05).
Bersamin et al., 2016	School‐based health center (SBHC)	California	High	Not reported	Not reported	Not reported	2012	Quasi‐experiment	Examine the association of SBHC presence with academic achievement and college preparation among California high school students	SBHC presence was positively associated with college preparation outcomes: ACT (B = 4.49, *p* < 0.01), AP (B = 5.65, *p* < 0.01), and SAT (B = 7.35, *p* < 0.01) participation rates. Analyses found no significant association between SBHC presence and graduation rates or meeting state university graduation requirements.
Gruber et al., 2023	School‐based health center (SBHC)	Michigan	High	413	Male: 45%–45.4% Female: 54.6%–55%	Black: 33.1%–38% White: 38%–43.6% Not Black or White: 22.5%–24.9%	2006–2009	Descriptive, longitudinal	Examine the effects of SBHC use on attendance among high school students	Analyses did not find that SBHC use directly (B < 0.01, SE < 0.01) nor indirectly (B < 0.01, SE < 0.01) predicted absenteeism through its effect on health or physical activity.
Long et al., 2024	Telehealth: school nurses and students virtually visit a medical provider	Maryland	Elementary	4203	Male: 52.2% Female: 47.8%	Non‐Hispanic White: 24.3% Non‐Hispanic Black: 45.1% Other Race/Ethnicity: 10.7% Hispanic: 19.7%	2014–2017	Quasi‐experiment	Evaluate the effectiveness of telehealth at reducing absences compared to standard school‐based wellness services	Absences were 7%–8% higher in students not enrolled in telehealth in 2015–2016 (7.20 vs. 6.64 days) and 2016–2017 (7.81 vs. 7.32). In the adjusted negative binomial regression model, students enrolled in the telehealth program were absent 7.7% (*p* = 0.025; 95% CI: 1.0%, 14%) fewer days per year than non‐telehealth students.
Dudovitz et al., 2020	Vision services: free screening, eye exam, and glasses	Los Angeles, California	Elementary	23,799	Male: 45.6%	African American: 18% Latino: 76%	2013–2016	Quasi‐experiment	Test whether a free school‐based vision‐care program was associated with improved school performance for low‐income minority students in L.A.	Results revealed no significant change in math achievement rank or work habits rank over the 2 years after receiving glasses. Students demonstrated improved ELA achievement rank by 4.5 percentile points in the second year after receiving glasses (*p* = 0.02).
Neitzel et al., 2021	Vision services: free screening, eye exam, and glasses	Baltimore, Maryland	Elementary Middle	2304	Female: 54.7%	Black: 77.6% White: 18.8% Asian: 1.4% Latinx: 16.8%	2015–2019	Experiment	Assess the effect of a school‐based vision program on academic achievement among students in grades 3–7	The vision program was associated with higher reading score (ES = 0.09; *p* = 0.02) and math scores among elementary students (ES = 0.03; *p* < 0.001) during school year 2016–2017. The intervention did not show a sustained impact at 2 years for college or career testing.
Hark et al., 2020	Vision services: free screening, eye exam, and glasses	The School District of Philadelphia, Pennsylvania	Elementary	349–4523	Girls: 48.7% Boys: 50.7% Unknown: 0.6%	Asian: 30.7% African American: 29.2% White: 18.9% Multiracial/other: 9.8% Hispanic: 11.5%	2014–2015	Quasi‐experiment	Assess the impact of eyeglass administration after a vision‐screening program on standardized testing scores in school‐aged children	Initially high reading performances were less likely to decline among students who received glasses compared to children who either passed their vision screen (with or without glasses they had) or children who failed their screen and did not receive glasses (OR = 4.36, *p* < 0.001). Analyses did not find an effect of the intervention for children with initially moderate or low reading performances.
Glewwe et al., 2018	Vision services: free screening, eye exam, and glasses	Central Florida	Elementary	14,572–15,422	Female: 49%	White: 26.4% Asian: 2.2% Black: 28.2% Multiracial: 3.4% Hispanic: 35.8%	2010–2013	Experiment	Quantify the impact of vision screening, accompanied by eye exams and eyeglasses on student outcomes, including test scores, attendance rates, and discipline incidents.	Students in full‐treatment schools were 2.0 percentage points more likely to be proficient in reading than their peers in control schools (*p* < 0.01). Analyses did not find differences for math scores, the total number of absences, unexcused absences, nor the number of behavioral referrals or suspensions between students in the full‐treatment schools and control schools.
Slavin et al., 2018	Vision services: free screening, eye exam, and glasses	Baltimore, Maryland	Elementary	262–317	Not reported	African American: 84.5% White: 4.4% Hispanic: 8.5%	2014–2015	Quasi‐experiment	Assess the effect of providing eyeglasses on the reading performance of disadvantaged students	Students who received glasses improved more on reading scores than those who never needed glasses (ES = 0.16, *p* < 0.05).

Abbreviations: ACT, American College Test; AIAN, American Indian and Alaska Native; AP, Advanced Placement; CI, confidence interval; DID, difference‐in‐difference; ELA, English Language Arts; ES, effect size; GPA, grade point average; Mod., moderate; NHPI, Native Hawaiian or Pacific Islander; OR, odds ratio; RCT, randomized controlled trial; SBHC, school‐based health center; SD, standard deviation; SE, standard error.

### 3.4 Evaluated Outcomes

From the 55 included studies, we identified three behavioral and learning outcome groups: school participation (i.e., attendance and school engagement), school performance (i.e., academic assessments [test scores, grades], graduation, and grade repeats), and school safety (i.e., disciplinary involvement, physical fighting or bullying, and substance use). Most included studies focused on multiple outcomes and outcome groups (e.g., evaluating universal free breakfast on attendance [school participation outcome] and academic assessment [school performance outcome]).

### 3.5 Evidence Map

**FIGURE 2:**
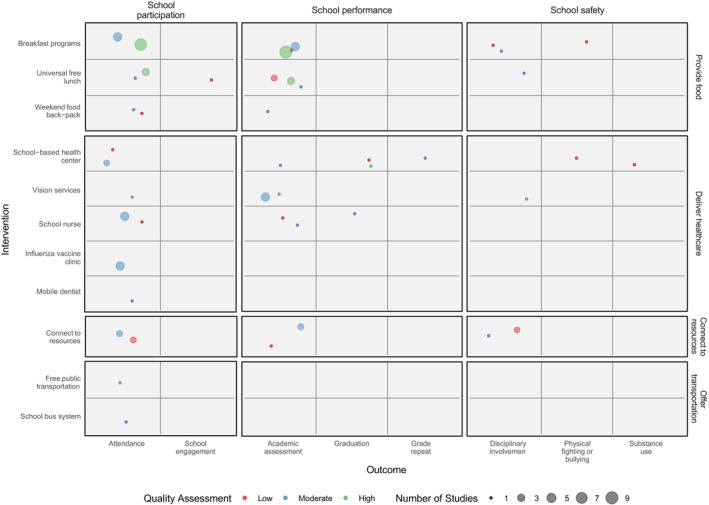
Evaluations of school‐based interventions to address unmet material needs according to evaluated outcomes. Breakfast programs include the School Breakfast Program, universal free breakfast, and breakfast after the bell. Universal free lunch includes both universal free meal programs and universal free lunch programs.

Figure 2 provides an overview of the studies in each intervention strategy‐outcome combination and the quality of such evidence. The most common was evaluating the effects of providing food on school performance (*n* = 21), followed by its effects on school participation (*n* = 19). Evaluating the effects of delivering healthcare on school participation (*n* = 14) and school performance (*n* = 10) were the next most common study combinations. Study combinations involving interventions to connect families to resources or offer transportation, or outcomes of school safety, were least common. Specifically, four studies evaluated the effects of connecting families to resources on school participation, and another four evaluated the effects of providing food on school safety. Three studies evaluated the effects of connecting families to resources on school performance or the effects of connecting families to resources on school safety. Only two studies evaluated the effects of delivering healthcare on school safety, and only two evaluated the effects of offering transportation on school participation. No studies evaluated the effects of offering transportation on school performance or school safety. Below, we organize findings by combinations of intervention type and outcome group.

**TABLE 3:** Study characteristics and findings for school‐based interventions to connect students or their families to resources and interventions to offer transportation.

Study	Intervention	Location	School level	Student *n*	Student gender	Student race and ethnicity	Study years	Study design	Study aims	Results
Meloche et al., 2020	RESOURCE CONNECTION: Community school and refugee center programming—school/agency partnerships that provide students and families with education, health, and social services	Sansom Valley School District, Pennsylvania	Middle	3426	Female: 48.2% Male: 51.8%	African American: 20.3% Asian (not Hispanic): 6.6% Multiracial (not Hispanic): 3.1% White: 15.4% Hispanic: 54.6%	2014–2016	Quasi‐experiment	Examine the impact of a community school and refugee center on immigrant and refugee youths' outcomes	Analyses did not find differences in attendance rates between students in treatment and comparison schools (*p*s > 0.05). English Language Learner students in treatment schools had fewer suspensions, and higher GPA, ELA, math, and science grades than those in comparison schools (*p*s < 0.05). 8th grade English Language Learner students in treatment schools had a lower proportion of students with a GPA ≥ 3.0 (25.7 vs. 11.4%), a lower proportion of students failing ELA (12.5 vs. 18.4%), a lower proportion of students failing math (6.3 vs. 24.0%), and a lower proportion of students with behavioral incidents (18.9 vs. 30.0%) than students in comparison schools (*p*s < 0.05).
Biag & Castrechini, 2016	RESOURCE CONNECTION: Community‐School Program, including support services (counseling, case management, family assistance [food aid, transport support, gift cards])	Redwood City School District—Bay Area, California	Elementary Middle Mixed level	5003	Male: 51.7% Female: 48.3%	Caucasian: 2.9% Latino: 91.6% Other ethnicity: 4%	2006–2013	Descriptive, longitudinal	Investigate the association between the community‐school programming and attendance and achievement	Use of support services was linked to lower attendance rates (B = −0.14, SE = 0.07; *p* = 0.03) and lower ELA achievement (B = −0.03, SE = 0.01, *p* = 0.05). Analyses found no association between use of social support services and math achievement (B = −0.02, SE = 0.02; *p* = 0.14).
Pullmann et al., 2013	RESOURCE CONNECTION: Family support workers connect students and families to academic support services and community resources	Seattle, Washington	Elementary	25,435	Male: 50.7% Female: 49.3%	White: 44.7% Asian: 16.9% Native American: 1% Black: 17.9% Multiracial: 6.2% NHPI: 0.5% Hispanic: 12.9%	2009–2011	Descriptive, longitudinal	Evaluate associations between student participation in a school‐based family support program and student outcomes	Qualitative data from focus groups indicated that school faculty/staff, families, and family support workers believe the program has a positive impact on academic functioning. Quantitative analyses did not find that the program was related to improvements in student attendance, disciplinary actions or standardized test scores.
Bates et al., 2019	RESOURCE CONNECTION: Interprofessional CARE teams assess student needs and provide interventions, such as dental and health services and family‐based supports	Large school district in the inter‐mountain west	Elementary	241–340	Male: > 65%	African American: 5% White or Caucasian: 49% Native American: 1% NHPI: 1% Multiracial: 5% Hispanic: 39%	2015–2016	Descriptive, longitudinal	Explore how interprofessional team collaboration affects student‐ outcomes	Analyses found no difference in absences before versus after CARE in the full sample (*p* = 0.65). Among 100 high‐risk students, students missed fewer days in the 8 weeks following service initiation (M = 4.43, SD = 3.79) compared to the 8 weeks prior (M = 5.48, SD = 3.22, *p* = 0.07), and had fewer referrals in the 8 weeks following (M = 0.68, SD = 1.12), compared to the 8 weeks prior (M = 1.38, SD = 0.99, *p* < 0.01). Analyses found no difference in disciplinary referrals before versus after CARE (*p* = 0.92).
Gottfried, 2017	TRANSPORTATION: School bus system	United States	Elementary	11,100–14,370	Male: 51.5%	Black: 13.7% Asian: 3.8% Other: 5.2% Hispanic: 25.3%	2010–2011	Cross‐sectional	Identify if kindergartners who took the bus to school had fewer total absences and lower chances of being chronically absent compared with those who went to school by other means	Children who took the bus to school had fewer absent days (B = −0.28, SE = 0.10) and were less likely to be chronically absent (B = −0.02, SE = 0.01) than students who traveled to school via other means.
Munoz and Sandoval, 2022	TRANSPORTATION: Free public transportation for students	Leon County, Florida	Middle High	Not reported	Not reported	African American: 43.6% White: 42.1% Hispanic: 6%	2003–2019	Quasi‐experiment	Estimate the effect of free bus fare on the school attendance behavior of middle and high school students	The free public transportation program was associated with a decrease of around 1 percentage point in school attendance and an increase of 2–4 percentage points in chronic absenteeism.

Abbreviations: ELA, English Language Arts; GPA, grade point average; Mod., moderate; NHPI, Native Hawaiian or Pacific Islander; SD, standard deviation; SE, standard error.

#### 3.5.1 The Effects of Providing Food on School Performance

The 21 studies that evaluated the effects of providing food on school performance all focused on academic assessment (e.g., test scores, grades) with mixed findings. Several studies (*n* = 11) found that school performance improved [30, 31, 32, 33, 34, 35, 36, 37, 38, 39, 40], with most studies finding divergent results among student subgroups (e.g., male versus female students) or assessment outcomes (e.g., grades versus standardized test scores). A similar number of analyses (*n* = 10) did not find effects, in either the full sample or subgroups [41, 42, 43, 44, 45, 46, 47, 48, 49, 50]. Three studies found that academic performance declined with these interventions for some subgroups [31, 32, 49].

#### 3.5.2 The Effects of Providing Food on School Participation

Among the 19 studies that evaluated the effects of providing food on school participation, most focused on school attendance and one focused on school engagement, with mixed findings. Most analyses (*n* = 10) found positive effects on attendance [30, 31, 34, 42, 43, 47, 51, 52, 53, 54], although almost an equal number (*n* = 8) did not find effects in the full sample [35, 36, 37, 39, 44, 48, 49, 55] or among student subgroups [34]. Only one analysis found that attendance worsened following the intervention [37].

#### 3.5.3 The Effects of Delivering Healthcare on School Participation

Among the 14 studies that evaluated the effects of delivering healthcare on school participation, all focused on school attendance with largely positive findings, overall. Most analyses (*n* = 9) found positive effects on attendance [56, 57, 58, 59, 60, 61, 62, 63, 64], with a few studies (*n* = 5) not finding any effects [65, 66, 67, 68, 69]. notably, all studies that involved influenza vaccine clinics found positive effects on school attendance [56, 59, 62, 63], as did five out of six studies involving school nurses [57, 58, 60, 61, 64].

#### 3.5.4 The Effects of Delivering Healthcare on School Performance

Among the ten studies that evaluated the effects of delivering healthcare on school performance, nine found that school performance improved for at least some students according to at least one metric [57, 58, 65, 69, 70, 71, 72, 73, 74]. These metrics included academic assessment (*n* = 9), graduation (*n* = 1), and grade repeats (*n* = 1). a few studies found different results among student subgroups (e.g., initially high versus low proficiency readers) or assessment outcomes (e.g., math versus english scores) [65, 71, 72, 73].

#### 3.5.5 The Effects of Connecting Families to Resources on School Participation, School Performance, and School Safety

Among the four studies that evaluated the effects of connecting families to resources on school participation, all focused on attendance and most (*n* = 3) did not find effects in the full sample [75, 76, 77]. One study, however, found that attendance improved following the intervention among high‐risk students [75]. One study found that attendance decreased after participating in the intervention [78].

The three studies that evaluated the effects of connecting families to resources on school performance all focused on academic assessment but had discrepant results, finding an increase [76], decrease [78], and no change [77] in academic assessments following the intervention.

Findings for disciplinary involvement were similarly mixed, with one study finding that disciplinary involvement decreased among English Language Learners [76], and two studies not finding any associations with disciplinary involvement [75, 77] following an intervention.

#### 3.5.6 The Effects of Providing Food and Delivering Healthcare on School Safety

All four studies that evaluated the effects of providing food on school safety found that these interventions were associated with a decrease in infractions [40, 54, 79, 80], but some found different effects by outcome (e.g., decreases for misconduct infractions but not aggressive behavior) [79] or among student subgroups (e.g., finding effects for white male elementary students, only) [80].

The two studies that evaluated the effects of interventions providing healthcare on school safety did not find any effects [65, 81].

#### 3.5.7 The Effects of Providing Transportation on School Participation

The two studies focused on providing transportation found opposite effects on school attendance, but for different populations. A cross‐sectional analyses found better attendance among elementary students who took the bus compared to other transportation means to school [82], whereas a quasi‐experimental analysis found that offering free public transportation to middle and high school students decreased attendance [83].

## 4 Discussion

In this scoping review, we mapped four intervention types within the broad category of addressing students' unmet material needs: providing food, delivering healthcare, connecting families to resources, and offering transportation. Similarly, we identified three outcome groups for which researchers have evaluated these interventions: school participation, school performance, and school safety. Overall, this field of research in the US has focused on the effects of providing food and delivering healthcare on school participation and performance; we know less about the effects of these interventions on school safety. Researchers have conducted comparatively less work on interventions connecting families to resources and providing students with transportation.

Considering fundamental cause theory, insufficient resources for meeting basic needs are upstream precursors to a range of poor outcomes for students [84]. Per this theory, unmet material needs are fundamental causes, as they are associated with multiple different student behavioral and learning outcomes, and these relationships likely exist through multiple mechanisms [85, 86, 87]. Based on the findings of this scoping review, the research literature is growing in its attention to unmet material needs as a fundamental cause of school participation and performance. Although there are many evaluations of interventions addressing more proximal determinants of school participation (e.g., attendance reports mailed to parents) [88] and school performance (e.g., tutoring) [89], the research literature is evolving to better understand how intervening on fundamental causes—including access to food and healthcare—influences student outcomes, including student attendance, grades, test scores, and graduation [31, 33, 58, 59, 60].

The field has not observed the same evolution in considering unmet material needs as fundamental causes of school violence. Yet, given evidence that unmet material needs increase the likelihood that a student experiences bullying [7, 8, 90], they may be root determinants of school violence. The mixed findings from the small number of studies incorporating violence‐related outcomes suggest that addressing material needs may improve school safety [40, 54, 65, 79, 80, 81]. Additional studies that rigorously evaluate the effects of addressing students' material needs on school violence would inform our understanding of universal strategies to improve school safety.

Concentrating the evidence on providing food may be a product of how schools, districts, and states implement such interventions [91, 92, 93]. Many strategies providing food to students—including extensions to the School Breakfast Program and National School Lunch Program—are national programs, implemented consistently for several years, with multiple treated units, and sometimes with staggered implementation. With the availability of a pre‐treatment period as well as comparison units (e.g., untreated schools), these interventions create opportunities for strong quasi‐experimental study designs [94]. For example, the Community Eligibility Provision (CEP) is an option for schools to offer meals at no cost to all enrolled students without collecting household applications [95]. As schools implement universal free meals through CEP, researchers gain the opportunity to study this natural experiment through quasi‐experimental analytic methods [30, 34, 38, 51].

The present evidence base for providing food to students is largely mixed between null and positive findings for its effects on school participation and performance. This is potentially a result of heterogeneous treatment effects between students. Specifically, several studies noted greater intervention effects for students living in households with lower annual incomes [32, 37, 38, 51]. Future analyses that investigate under which conditions and for whom these interventions improve outcomes may clarify this evidence base and suggest mechanisms through which providing school meals influences student learning and behavior. The mixed findings within this intervention type may also be a product of distinct intervention strategies (making food more available [i.e., BATB] vs. making food more accessible [i.e., UFL/UFM]). Although obtaining a causal estimate is outside the scope of this review, future meta‐analyses might consider these distinctions when evaluating the effects of interventions providing food.

The present evidence base for delivering healthcare to students is largely positive for its effects on school participation and performance; most studies that evaluated effects on school participation, and nearly all studies that evaluated effects on school performance, found positive effects. This consistency might stem from how healthcare interventions, such as providing glasses or vaccinations, can produce immediate results with small time commitments from participants.

Interventions connecting families to resources may be more difficult to rigorously evaluate compared to providing food or delivering healthcare. These interventions often depend on relationships between school and community partners as well as inter‐professional relationships [96]. These relationships, by nature, are dynamic and vary in quality and quantity between schools and over time [97]. Although this relational nature can benefit students and their families, it can also make implementing interventions with fidelity across schools and over time difficult, thereby complicating evaluation efforts. However, recent evaluations of community‐based family resource centers might offer a promising approach that explicitly accounts for local adaptation while maintaining core components that can be evaluated across sites [98], thereby building more robust evidence while honoring the flexible and relationship‐based nature of these interventions.

Most analyses focused on school participation and school performance outcomes, with comparatively less focus on school safety [99]. This may be due to the availability and standardized nature (across schools and time) of metrics capturing school participation and performance, including attendance records and standardized test scores. Although educational entities routinely collect and report some school safety outcomes, including school expulsions and suspensions, there are substantial biases in these data due to racial inequities in how schools apply exclusionary discipline [100]. Further, reporting varies by state, with many states only reporting expulsions, and other states having no reporting requirements for suspensions or expulsions [101]. Schools rarely collect and report data on less severe forms of school violence, such as isolation and bullying.

### 4.1 Implications for School Health Policy, Practice, and Equity

School‐based interventions to address students and their families' unmet material needs may improve student outcomes. The present evidence suggests that school‐based healthcare, in particular, can benefit school performance and participation. Several programmatic and policy structures in the US can facilitate school‐based healthcare [102]. For example, 28 states have expanded their school Medicaid program to cover services outside of an Individualized Education Plan as of March 2026 [103], which research suggests increases the number of centers providing school‐based services [104]. With respect to building health and educational equity, researchers have also found that school‐based healthcare increases access for rural communities [105] and reduces income‐based health disparities [106].

The evidence for providing food on school performance and participation is mixed. Further, the field is underdeveloped for interventions connecting families to resources or offering transportation, and for the effects of any of these material needs interventions on school safety. Yet, fundamental cause theory argues that strategies to improve resource access and basic social conditions can efficiently improve a range of outcomes, and this may extend to academic performance, engagement, and behavior. As such, school‐based interventions to address unmet student needs may be an equitable intervention model to promote overall student and school wellbeing. Future, high‐quality studies that investigate when and for whom these interventions are effective can guide policy and implementation efforts.

### 4.2 Limitations

Per JBI scoping review methodological guidance [27], we aimed to be as comprehensive as possible within the constraints of time, resources, and topic. This topic has a large and interdisciplinary breadth, and so our search may have missed studies. Yet, the literature identified effectively represents and maps the breadth of research on this topic. Additionally, this review focused on general K‐12 education settings, and it did not include studies focused exclusively on students with disabilities or alternative learning institutions. Further, the review excluded non‐US studies, thereby limiting generalizability. Finally, as a scoping review, we did not aim to obtain causal estimates. In defining the different intervention types and outcome groups in this field, however, our review can inform future meta‐analyses.

## 5 Conclusions

Evaluating school‐based interventions that address students and their families' unmet material needs is a distinct and interdisciplinary area of research. Promising evidence exists for the effects of delivering healthcare on improving school participation and performance. The evidence for providing food on these outcomes is mixed, and future studies that investigate when and for whom these interventions are effective can guide implementation efforts. Finally, the field is underdeveloped for interventions connecting families to resources or offering transportation, and for the effects of material needs interventions on school safety. Future studies that consider the multiple effects of these school‐based interventions on school performance, participation, and safety can potentially offer efficient and effective solutions to schools' most pressing issues.

## Funding

This work was supported by the Centers for Disease Control and Prevention (R01‐CE003536).

## Ethics Statement

Preparation of this paper did not involve primary research or data collection involving human subjects, and therefore, no institutional review board examination or approval was required.

## Conflicts of Interest

The authors declare no conflicts of interest.

## Supporting information


**METHODS S1:** Database search queries.
**TABLE S1:** Intervention characteristics of included studies (*n* = 55).
**TABLE S2:** Study characteristics and findings of included studies (*n* = 55).
**TABLE S3:** Settings and populations of included studies (*n* = 55).
**TABLE S4:** Quality assessment for qualitative studies (*n* = 1).
**TABLE S5:** Quality assessment for randomized controlled trials (*n* = 5).
**TABLE S6:** Quality assessment for nonrandomized quantitative studies (*n* = 49).
**RESULTS S1:** Evidence quality.

## Data Availability

Data sharing not applicable to this article as no datasets were generated or analysed during the current study.
