# Supplementary material for: School‐Based Interventions That Address Students' Unmet Material Needs: A Scoping Review
Source: J Sch Health. 2026 Aug 9;96(9):e70207. doi: 10.1111/josh.70207 (PMC13453552; doi:10.1111/josh.70207)
Supplement: Supplementary file 1 [file JOSH-96-0-s001.doc]

# Supplement

School-based interventions that address students’ unmet material needs: A scoping review

Contents

**Supplemental Methods 1**. Database search queries	2

**Supplemental Table 1**. Intervention characteristics of included studies (n=55).	8

**Supplemental Table 2**. Study characteristics and findings of included studies (n=55).	10

**Supplemental Table 3**. Settings and populations of included studies (n=55).	16

**Supplemental Table 4**. Quality assessment for qualitative studies (n=1).	21

**Supplemental Table 5**. Quality assessment for randomized controlled trials (n=5).	22

**Supplemental Table 6**. Quality assessment for non-randomized quantitative studies (n=49).	23

**Supplemental Results 1.** Evidence quality	25

# **Supplemental Methods 1**. Database search queries

| **Database search queries** | | | | | | |
| --- | --- | --- | --- | --- | --- | --- |
| **Database** | **Filter** | **Criteria 1** | **Criteria 2** | **Criteria 3** | **Criteria 4** | **Final** |
| ERIC (ProQuest)  Results: 6,885 | Schol-arly journals | MAINSUBJECT.EXACT("Schools") OR title(school* or kindergarten* OR aftercare OR after-care OR “junior high”) OR abstract(school* OR kindergarten* OR aftercare OR after-care OR “junior high”) | ((MAINSUBJECT.EXACT"Health Programs") OR MAINSUBJECT.EXACT("Breakfast Programs") OR MAINSUBJECT.EXACT("Lunch Programs") OR MAINSUBJECT.EXACT("Immunization Programs") OR MAINSUBJECT.EXACT("Family Programs") OR MAINSUBJECT.EXACT("Family Income") OR MAINSUBJECT.EXACT.EXPLODE("Transportation") OR MAINSUBJECT.EXACT("Bus Transportation") OR MAINSUBJECT.EXACT("Student Transportation") OR MAINSUBJECT.EXACT("Human Services") OR MAINSUBJECT.EXACT.EXPLODE("Integrated Services") OR MAINSUBJECT.EXACT("Welfare Services") OR MAINSUBJECT.EXACT.EXPLODE("Food Service") OR MAINSUBJECT.EXACT("School Health Services") OR MAINSUBJECT.EXACT("Prenatal Care") OR MAINSUBJECT.EXACT.EXPLODE("Medical Services") OR MAINSUBJECT.EXACT("Health Services") OR MAINSUBJECT.EXACT("Assistive Technology") OR MAINSUBJECT.EXACT("Clothing") OR MAINSUBJECT.EXACT("Resources") OR MAINSUBJECT.EXACT("Housing Needs") OR MAINSUBJECT.EXACT("Utilities")) AND (title(assistance OR aid OR aids OR policy OR policies OR program* OR service OR services OR provision* OR providing OR provide OR receipt OR intervention* OR project OR projects OR pilot OR pilots OR center OR centers) OR abstract(assistance OR aid OR aids OR policy OR policies OR program* OR service OR services OR provision* OR providing OR provide OR intervention* OR project OR projects OR pilot OR pilots OR center OR centers)) | title("Material need*" OR "material support*" OR "material hardship*" OR "financial hardship*" OR "public benefit*" OR ((integrated OR medical OR medicine OR medication OR health OR healthcare OR health-care OR "health care" OR under-insured OR underinsured OR "under insured" OR un-insured OR uninsured OR medicaid OR immuniz* OR immunis* OR dental OR dentistry OR vision OR glasses OR eyeglasses OR “hearing aid*” OR wheelchair* OR toiletry OR toiletries OR welfare OR housing OR house OR houses OR homeless* OR unhoused OR shelter OR shelters OR lodging OR transport* OR car OR cars OR "motor vehicle*" OR automobile* OR bus OR busses OR busing OR taxi OR food OR hunger OR hungry OR meal OR meals OR breakfast* OR lunch* OR nutrition* OR dietary OR snack OR snacks OR fruit* OR vegetable* OR SNAP OR WIC OR "school supply" OR "school supplies" OR backpack* OR pen OR pens OR pencil* OR paper OR clothing OR clothes OR garment* OR coat OR coats OR shoe OR shoes OR uniform* OR poverty OR poor OR low-income OR "low income" OR disadvantaged OR financial OR economic OR cash OR utilities OR electricity OR gas) NEAR/3 (assistance OR aid OR aids OR policy OR policies OR program* OR service OR services OR provision* OR providing OR provide OR intervention* OR project OR projects OR pilot OR pilots OR center OR centers)) ) OR abstract("Material need*" OR "material support" OR "material hardship*" OR "financial hardship*" OR "public benefit*" OR ((integrated OR medical OR medicine OR medication OR health OR healthcare OR health-care OR "health care" OR under-insured OR underinsured OR "under insured" OR un-insured OR uninsured OR medicaid OR immuniz* OR immunis* OR dental OR dentistry OR vision OR glasses OR eyeglasses OR “hearing aid*” OR wheelchair* OR toiletry OR toiletries OR welfare OR housing OR house OR houses OR homeless* OR unhoused OR shelter OR shelters OR lodging OR transport* OR car OR cars OR "motor vehicle*" OR automobile* OR bus OR busses OR busing OR taxi OR food OR hunger OR hungry OR meal OR meals OR breakfast* OR lunch* OR nutrition* OR dietary OR snack OR snacks OR fruit* OR vegetable* OR SNAP OR WIC OR "school supply" OR "school supplies" OR backpack* OR pen OR pens OR pencil* OR paper OR clothing OR clothes OR garment* OR coat OR coats OR shoe OR shoes OR uniform* OR poverty OR poor OR low-income OR "low income" OR disadvantaged OR Financial OR economic OR cash OR utilities OR electricity OR gas) NEAR/3 (assistance OR aid OR aids OR policy OR policies OR program* OR service OR services OR provision* OR providing OR provide OR intervention* OR project OR projects OR pilot OR pilots OR center OR centers))) | title((family OR families) NEAR/3 (income OR incomes OR wage OR wages OR salary OR salaries OR earning OR earnings)) OR abstract((family OR families) NEAR/3 (income OR incomes OR wage OR wages OR salary OR salaries OR earnings OR earning)) OR title((internet OR wifi OR wi-fi OR wireless) NEAR/3 (assistance OR aid OR aids OR policy OR policies OR program* OR service OR services OR provision* OR intervention* OR project OR projects OR pilot OR pilots OR center OR centers)) OR abstract((internet OR wifi OR wi-fi OR wireless) NEAR/3 (access* OR avail* OR assistance OR aid OR aids OR policy OR policies OR program* OR service OR services OR provision* OR intervention* OR project OR projects OR pilot OR pilots OR center OR centers)) | 1 & (2 OR 3 OR 4) |
| Sociol. Abstracts (ProQuest)  Results: 2,546 | Schol-arly journals | MAINSUBJECT.EXACT("Schools") OR MAINSUBJECT.EXACT("Elementary schools") OR MAINSUBJECT.EXACT("Public schools") OR MAINSUBJECT.EXACT("Private schools") OR MAINSUBJECT.EXACT("Secondary schools") OR MAINSUBJECT.EXACT("Middle schools") OR MAINSUBJECT.EXACT("Kindergarten") OR title(school* or kindergarten OR aftercare or after-care or "junior high" ) OR abstract(school* or kindergarten OR aftercare or after-care or "junior high") | (MAINSUBJECT.EXACT("Health services") OR MAINSUBJECT.EXACT("Family Income") OR MAINSUBJECT.EXACT.EXPLODE("Transportation") OR MAINSUBJECT.EXACT("Human Services") OR MAINSUBJECT.EXACT("Welfare services") OR MAINSUBJECT.EXACT("Social services") OR MAINSUBJECT.EXACT("Food security") OR MAINSUBJECT.EXACT("Prenatal Care") OR MAINSUBJECT.EXACT("Clothing") OR MAINSUBJECT.EXACT("Resources")) AND (title(assistance OR aid OR aids OR policy OR policies OR program* OR service OR services OR provision* OR providing OR provide OR receipt OR intervention* OR project OR projects OR pilot OR pilots OR center OR centers) OR abstract(assistance OR aid OR aids OR policy OR policies OR program* OR service OR services OR provision* OR providing OR provide OR intervention* OR project OR projects OR pilot OR pilots OR center OR centers)) | title("Material need*" OR "material support*" OR "material hardship*" OR "financial hardship*" OR "public benefit*" OR ((integrated OR medical OR medicine OR medication OR health OR healthcare OR health-care OR "health care" OR under-insured OR underinsured OR "under insured" OR un-insured OR uninsured OR medicaid OR immuniz* OR immunis* OR dental OR dentistry OR vision OR glasses OR eyeglasses OR “hearing aid*” OR wheelchair* OR toiletry OR toiletries OR welfare OR housing OR house OR houses OR homeless* OR unhoused OR shelter OR shelters OR lodging OR transport* OR car OR cars OR "motor vehicle*" OR automobile* OR bus OR busses OR busing OR taxi OR food OR hunger OR hungry OR meal OR meals OR breakfast* OR lunch* OR nutrition* OR dietary OR snack OR snacks OR fruit* OR vegetable* OR SNAP OR WIC OR "school supply" OR "school supplies" OR backpack* OR pen OR pens OR pencil* OR paper OR clothing OR clothes OR garment* OR coat OR coats OR shoe OR shoes OR uniform* OR poverty OR poor OR low-income OR "low income" OR disadvantaged OR financial OR economic OR cash OR utilities OR electricity OR gas) NEAR/3 (assistance OR aid OR aids OR policy OR policies OR program* OR service OR services OR provision* OR providing OR provide OR intervention* OR project OR projects OR pilot OR pilots OR center OR centers)) ) OR abstract("Material need*" OR "material support" OR "material hardship*" OR "financial hardship*" OR "public benefit*" OR ((integrated OR medical OR medicine OR medication OR health OR healthcare OR health-care OR "health care" OR under-insured OR underinsured OR "under insured" OR un-insured OR uninsured OR medicaid OR immuniz* OR immunis* OR dental OR dentistry OR vision OR glasses OR eyeglasses OR “hearing aid*” OR wheelchair* OR toiletry OR toiletries OR welfare OR housing OR house OR houses OR homeless* OR unhoused OR shelter OR shelters OR lodging OR transport* OR car OR cars OR "motor vehicle*" OR automobile* OR bus OR busses OR busing OR taxi OR food OR hunger OR hungry OR meal OR meals OR breakfast* OR lunch* OR nutrition* OR dietary OR snack OR snacks OR fruit* OR vegetable* OR SNAP OR WIC OR "school supply" OR "school supplies" OR backpack* OR pen OR pens OR pencil* OR paper OR clothing OR clothes OR garment* OR coat OR coats OR shoe OR shoes OR uniform* OR poverty OR poor OR low-income OR "low income" OR disadvantaged OR Financial OR economic OR cash OR utilities OR electricity OR gas) NEAR/3 (assistance OR aid OR aids OR policy OR policies OR program* OR service OR services OR provision* OR providing OR provide OR intervention* OR project OR projects OR pilot OR pilots OR center OR centers))) | title((family OR families) NEAR/3 (income OR incomes OR wage OR wages OR salary OR salaries OR earning OR earnings)) OR abstract((family OR families) NEAR/3 (income OR incomes OR wage OR wages OR salary OR salaries OR earnings OR earning)) OR title((internet OR wifi OR wi-fi OR wireless) NEAR/3 (assistance OR aid OR aids OR policy OR policies OR program* OR service OR services OR provision* OR providing OR provide OR intervention* OR project OR projects OR pilot OR pilots OR center OR centers)) OR abstract((internet OR wifi OR wi-fi OR wireless) NEAR/3 (access* OR avail* OR assistance OR aid OR aids OR policy OR policies OR program* OR service OR services OR provision* OR providing OR provide OR intervention* OR project OR projects OR pilot OR pilots OR center OR centers)) | 1 & (2 OR 3 OR 4) |
| Education Abstracts  (EBSCOhost)  Results: 2,979 | Schol-arly journals | DE "Schools" OR TI ( school* OR kindergarten OR aftercare OR after-care OR “junior high” ) OR AB ( school* OR kindergarten OR after-care OR “junior high” ) | (DE "Health programs" OR DE "Family services" OR DE "Immunization" OR DE "School breakfast programs" OR DE "Health education" OR DE "Transportation of school children" OR DE "Busing for school integration" OR DE "School bus companies" OR DE "School buses"OR DE "Human services programs" OR DE "After school programs" OR DE "Developmental programs" OR DE "School food" OR DE "National school lunch program" OR DE "School breakfast programs" OR DE "School milk programs" OR DE "School health services" OR DE "Coordinated school health programs" OR DE "School-linked human services" OR DE "Childbirth education" DE "Clothing & dress" OR DE "Education of welfare recipients" OR DE "Assistive technology" OR DE "Adapted teaching aids & devices" OR DE "Apparatus for the blind" OR DE "Assistive computer technology" OR DE "Communication devices for people with disabilities" OR DE "Reading devices for people with disabilities" OR DE "Clothing & dress" OR DE "School uniforms" OR DE "School supplies" OR DE "Blackboards" OR DE "Student housing") AND ( TI ( assist* OR aid OR aids OR policy OR policies OR program* OR service* OR provision* OR providing OR provide OR intervention* OR project* OR pilot* OR center* ) OR AB ( assist* OR aid OR aids OR policy OR policies OR program* OR service* OR provision* OR providing OR provide OR intervention* OR project* OR pilot* OR center* ) ) | (title(“Material need*” OR “material support*” OR “material hardship*” OR “financial hardship*” OR “public benefits” OR under-insured OR underinsured OR “under insured” OR un-insured OR uninsured OR ((integrated OR medical OR medicine OR OR medication OR health OR healthcare OR medicaid OR immuniz* OR immunis* OR dental OR dentistry OR vision OR glasses OR eyeglasses OR “hearing aid*” OR wheelchair* OR housing OR house OR houses OR homeless* OR unhoused OR shelter OR shelters OR lodging OR transport* OR car OR cars OR “motor vehicle*” OR automobile* OR bus OR busses OR busing OR taxi OR food OR hunger OR hungry OR meal OR breakfast* OR lunch* OR nutrition* OR dietary OR snack OR snacks OR fruit* OR vegetable* OR SNAP OR WIC OR “school supply” OR “school supplies” OR backpack* OR pen OR pens OR pencil* OR paper OR clothing OR clothes OR garment* OR coat OR coats OR shoe OR shoes OR uniform* OR non-academic OR equity OR inequity OR poverty OR poor OR low-income OR “low income” OR disadvantaged OR Financial OR economic OR cash OR utilities OR electricity OR gas) N3 (assist* OR aid OR aids OR policy OR policies OR program* OR service* OR provision* OR providing OR provide OR intervention* OR project* OR pilot* OR center*)) ) OR abstract(“Material need*” OR “material hardship*” OR “financial hardship” OR “public benefits” OR ((integrated OR medical OR medicine OR medication OR health OR healthcare OR under-insured OR underinsured OR “under insured” OR un-insured OR uninsured OR welfare OR immuniz* OR immunis* OR dental OR dentistry OR vision OR glasses OR eyeglasses OR “hearing aid*” OR wheelchair* OR housing OR house OR houses OR homeless* OR unhoused OR shelter OR shelters OR lodging OR transport* OR car OR cars OR “motor vehicle*” OR automobile* OR bus OR busses OR busing OR taxi OR food OR hunger OR hungry OR meal OR breakfast* OR lunch* OR nutrition* OR dietary OR snack OR snacks OR fruit* OR vegetable* OR SNAP OR WIC OR “school supply” OR “school supplies” OR backpack* OR pen OR pens OR pencil* OR paper OR clothing OR clothes OR garment* OR coat OR coats OR shoe OR shoes OR uniform* OR non-academic OR equity OR inequity OR poverty OR poor OR low-income OR “low income” OR disadvantaged OR Financial OR economic OR cash OR utilities OR electricity OR gas) N3 (assist* OR aid OR aids OR policy OR policies OR program* OR service* OR provision* OR providing OR provide OR intervention* OR project* OR pilot* OR center*)) ) ) | TI ( (( family OR families ) N3 ( income OR incomes OR wage OR wages OR salary OR salaries OR earning OR earnings ) ) OR ( ( internet OR wifi OR wi-fi OR wireless ) N3 ( access* OR avail* OR assist* OR aid OR aids OR policy OR policies OR program* OR service* OR provision* OR providing OR provide OR intervention* OR project* OR pilot* OR center* ) ) ) OR AB ( (( family OR families ) N3 ( income OR incomes OR wage OR wages OR salary OR salaries OR earning OR earnings ) ) OR ( ( internet OR wifi OR wi-fi OR wireless ) N3 ( access* OR avail* OR assist* OR aid OR aids OR policy OR policies OR program* OR service* OR provision* OR providing OR provide OR intervention* OR project* OR pilot* OR center* ) ) ) | 1 & (2 OR 3 OR 4) |
| PsycInfo  Results: 2,486 | Aca-demic Jour-nals | DE "Schools" OR DE "Boarding Schools" OR DE "Charter Schools" OR DE "Elementary Schools" OR DE "High Schools" OR DE "Junior High Schools" OR DE "Kindergartens" OR DE "Middle Schools" OR TI ( school* OR kindergarten OR aftercare OR after-care OR “junior high” ) OR AB ( school* OR kindergarten OR after-care OR “junior high” ) | ( DE "Health Care Services" OR DE "Family Socioeconomic Status" OR DE "Transportation" OR DE "Air Transportation" OR DE "Ground Transportation" OR DE "Public Transportation" OR DE "Water Transportation" OR DE "Air Transportation" OR DE "Ground Transportation" OR DE "Public Transportation" OR DE "Water Transportation" OR DE "Human Services" OR DE "Integrated Services" OR DE "Welfare Services (Government)" OR DE "Food Insecurity" OR DE "Prenatal Care" OR DE "Reproductive Health Care" OR DE "Clothing" OR DE "Housing" ) AND ( TI ( assist* OR aid OR aids OR policy OR policies OR program* OR service* OR provision* OR providing OR provide OR intervention* OR project* OR pilot* OR center* ) OR AB ( assist* OR aid OR aids OR policy OR policies OR program* OR service* OR provision* OR providing OR provide OR intervention* OR project* OR pilot* OR center* )) | (title(“Material need*” OR “material support*” OR “material hardship*” OR “financial hardship*” OR “public benefits” OR under-insured OR underinsured OR “under insured” OR un-insured OR uninsured OR ((integrated OR medical OR medicine OR OR medication OR health OR healthcare OR medicaid OR immuniz* OR immunis* OR dental OR dentistry OR vision OR glasses OR eyeglasses OR “hearing aid*” OR wheelchair* OR housing OR house OR houses OR homeless* OR unhoused OR shelter OR shelters OR lodging OR transport* OR car OR cars OR “motor vehicle*” OR automobile* OR bus OR busses OR busing OR taxi OR food OR hunger OR hungry OR meal OR breakfast* OR lunch* OR nutrition* OR dietary OR snack OR snacks OR fruit* OR vegetable* OR SNAP OR WIC OR “school supply” OR “school supplies” OR backpack* OR pen OR pens OR pencil* OR paper OR clothing OR clothes OR garment* OR coat OR coats OR shoe OR shoes OR uniform* OR non-academic OR equity OR inequity OR poverty OR poor OR low-income OR “low income” OR disadvantaged OR Financial OR economic OR cash OR utilities OR electricity OR gas) N3 (assist* OR aid OR aids OR policy OR policies OR program* OR service* OR provision* OR providing OR provide OR intervention* OR project* OR pilot* OR center*)) ) OR abstract(“Material need*” OR “material hardship*” OR “financial hardship” OR “public benefits” OR ((integrated OR medical OR medicine OR medication OR health OR healthcare OR under-insured OR underinsured OR “under insured” OR un-insured OR uninsured OR welfare OR immuniz* OR immunis* OR dental OR dentistry OR vision OR glasses OR eyeglasses OR “hearing aid*” OR wheelchair* OR housing OR house OR houses OR homeless* OR unhoused OR shelter OR shelters OR lodging OR transport* OR car OR cars OR “motor vehicle*” OR automobile* OR bus OR busses OR busing OR taxi OR food OR hunger OR hungry OR meal OR breakfast* OR lunch* OR nutrition* OR dietary OR snack OR snacks OR fruit* OR vegetable* OR SNAP OR WIC OR “school supply” OR “school supplies” OR backpack* OR pen OR pens OR pencil* OR paper OR clothing OR clothes OR garment* OR coat OR coats OR shoe OR shoes OR uniform* OR non-academic OR equity OR inequity OR poverty OR poor OR low-income OR “low income” OR disadvantaged OR Financial OR economic OR cash OR utilities OR electricity OR gas) N3 (assist* OR aid OR aids OR policy OR policies OR program* OR service* OR provision* OR providing OR provide OR intervention* OR project* OR pilot* OR center*)) ) ) | TI ( (( family OR families ) N3 ( income OR incomes OR wage OR wages OR salary OR salaries OR earning OR earnings ) ) OR ( ( internet OR wifi OR wi-fi OR wireless ) N3 ( access* OR avail* OR assist* OR aid OR aids OR policy OR policies OR program* OR service* OR provision* OR providing OR provide OR intervention* OR project* OR pilot* OR center* ) ) ) OR AB ( (( family OR families ) N3 ( income OR incomes OR wage OR wages OR salary OR salaries OR earning OR earnings ) ) OR ( ( internet OR wifi OR wi-fi OR wireless ) N3 ( access* OR avail* OR assist* OR aid OR aids OR policy OR policies OR program* OR service* OR provision* OR providing OR provide OR intervention* OR project* OR pilot* OR center* ) ) ) | 1 & (2 OR 3 OR 4) |
| Scopus  Results: 14,117 |  | TITLE(school* or kindergarten* OR aftercare OR after-care OR “junior high”) | TITLE-ABS({Human Services} OR {Prenatal Care} OR {Material need} OR {material needs} OR {Material support} OR {material supports} OR {Material hardship} OR {material hardships} OR {financial hardship} OR {financial hardships} OR {assistive technology} OR {assistive technologies} OR {public benefit} OR {public benefits} OR ((integrated OR medical OR medicine OR medication OR health OR healthcare OR health-care OR "health care" OR welfare OR under-insured OR underinsured OR "under insured" OR un-insured OR uninsured OR medicaid OR immuniz* OR immunis* OR dental OR dentistry OR vision OR glasses OR eyeglasses OR “hearing aid*” OR wheelchair* OR toiletry OR toiletries OR housing OR house OR houses OR homeless* OR unhoused OR shelter OR shelters OR lodging OR transport* OR car OR cars OR "motor vehicle*" OR automobile* OR bus OR busses OR busing OR taxi OR food OR hunger OR hungry OR meal OR breakfast* OR lunch* OR nutrition* OR dietary OR snack OR snacks OR fruit* OR vegetable* OR SNAP OR WIC OR "school supply" OR "school supplies" OR backpack* OR pen OR pens OR pencil* OR paper OR clothing OR clothes OR garment* OR coat OR coats OR shoe OR shoes OR uniform* OR poverty OR poor OR low-income OR "low income" OR disadvantaged OR financial OR economic OR cash OR utilities OR electricity OR gas) W/3 (assistance OR aid OR aids OR policy OR policies OR program* OR service* OR provision* OR providing OR provide OR intervention* OR project* OR pilot* OR center*))) | TITLE-ABS ( ( ( family OR families ) W/3 ( income OR incomes OR wage OR wages OR salary OR salaries OR earning OR earnings ) ) OR ( ( internet OR wifi OR wi-fi OR wireless ) W/3 ( access* OR avail* OR assist* OR aid OR aids OR policy OR policies OR program* OR service* OR provision* OR providing OR provide OR intervention* OR project* OR pilot* OR center* ) ) ) |  | 1 & (2 OR 3) |
| Medline (OVID)  Results: 9,725 | NOT: exp animals/ not humans.sh. | *schools/ OR (school* or kindergarten* or aftercare or after-care or "junior high").ti. | (*School Health Services/ or *child health services/ or *transportation/ or *motor vehicles/ or *automobiles/ or *social welfare/ or *food services/ or *public assistance/ or *health services/ or *prenatal care/ or *clothing/ or *housing instability/ or *housing/) AND (assistance OR aid OR aids OR policy OR policies OR program* OR service OR services OR provision* OR providing OR provide OR intervention* OR project OR projects OR pilot OR pilots OR center OR centers).ti,ab. | ("Material need*" or "material support" or "material hardship*" or "financial hardship*" or "public benefits").ti,ab. or ((integrated or medical or medicine OR medication or health or healthcare or health-care or under-insured or underinsured or "under insured" or un-insured or uninsured or medicaid or immuniz* or immunis* or dental OR dentistry OR vision OR glasses OR eyeglasses OR “hearing aid*” OR wheelchair* OR toiletry or toiletries or welfare or housing or house or houses or homeless* or unhoused or shelter or shelters or lodging or transport* or car or cars or "motor vehicle*" or automobile* or bus or busses or busing or taxi or food or hunger or hungry or meal or meals or breakfast* or lunch* or nutrition* or dietary or snack or snacks or fruit* or vegetable* or SNAP or WIC or "school supply" or "school supplies" or backpack* or pen or pens or pencil* or paper or clothing or clothes or garment* or shoe or shoes or coat or coats or uniform* or poverty or poor or low-income or "low income" or disadvantaged or financial or economic or cash or utilities or electricity or gas) adj3 (assistance OR aid OR aids OR policy OR policies OR program* OR service OR services OR provision* OR providing OR provide OR intervention* OR project OR projects OR pilot OR pilots OR center OR centers)).ti,ab. | ((family or families) adj3 (income or incomes or wage or wages or salary or salaries or earning or earnings)).ti,ab. or ((internet or wifi OR wi-fi or wireless) adj3 (access* or avail* or assistance OR aid OR aids OR policy OR policies OR program* OR service OR services OR provision* OR providing OR provide OR intervention* OR project OR projects OR pilot OR pilots OR center OR centers)).ab,ti. | 1 & (2 OR 3 OR 4) |

# **Supplemental Table 1**. Intervention characteristics of included studies (n=55).

| **Study** | **Intervention description** | **Intervention classification** | **Material needs addressed** |
| --- | --- | --- | --- |
| Meloche 2020 | Community school and refugee center programming - school/agency partnerships that provide students and families with education, health, and social services | Universal | Food; Healthcare (Connecting to resources) |
| Biag 2016 | Community-School Program, including social support services (counseling, case management services, family assistance [food aid, transportation supports, grocery gift cards]) | Universal | Transport; Food (Connecting to resources) |
| Pullmann 2013 | Family support workers connect students and families to academic support services and community resources | Selective; Indicated | Transport; Food; Clothing; School supplies; Healthcare; Housing (Connecting to resources) |
| Bates 2019 | Interprofessional CARE teams assess student needs and provide interventions within the school, such as dental and health services and family-based supports | Selective | Food; Healthcare; Housing (Connecting to resources) |
| Cuadros-Meñaca 2022a | Breakfast after the bell | Universal | Food |
| Abouk 2022 | Breakfast after the bell | Universal | Food |
| Cuadros-Meñaca 2023 | Breakfast after the bell | Universal | Food |
| Anzman-Frasca 2015 | Breakfast after the bell | Universal | Food |
| Gottfried 2022 | Breakfast after the bell | Universal | Food |
| Hearst 2019 | Breakfast after the bell | Universal | Food |
| Cuadros-Meñaca 2022b | Breakfast after the bell | Universal | Food |
| Kirksey 2021 | Breakfast after the bell | Universal | Food |
| Walker 2021 | Breakfast after the bell | Universal | Food |
| Corcoran 2016 | Breakfast after the bell | Universal | Food |
| Chandrasekhar 2023 | Breakfast after the bell | Universal; Indicated | Food |
| Luan 2022 | Breakfast after the bell; Universal Free Breakfast | Universal | Food |
| Imberman 2014 | Breakfast after the bell; Universal Free Breakfast | Universal | Food |
| Frisvold 2015 | School Breakfast Program | Universal | Food |
| Bartfeld 2019 | School Breakfast Program; Universal free breakfast; Breakfast after the bell | Universal; Indicated | Food |
| Ribar 2013 | Universal free breakfast | Universal; Indicated | Food |
| Bullock 2022 | Universal free breakfast | Universal | Food |
| Leos-Urbel 2013 | Universal free breakfast | Universal | Food |
| Williams 2022 | Universal free lunch | Universal | Food |
| Gordanier 2020 | Universal free lunch through the Community Eligibility Provision | Universal | Food |
| Schwartz 2020 | Universal free meals | Universal | Food |
| Taylor 2020 | Universal free | Universal | Food |
| Andreyeva 2021 | Universal free meals through the Community Eligibility Provision | Universal | Food |
| Ruffini 2022 | Universal free meals through the Community Eligibility Provision | Universal | Food |
| Gordon 2021 | Universal free meals through the Community Eligibility Provision | Universal | Food |
| Bartfeld 2020 | Universal free meals through the Community Eligibility Provision | Universal | Food |
| Kurtz 2020 | Weekend food back-pack (BackPack) | Selective | Food |
| Fiese 2020 | Weekend food back-pack (BackPack) | Selective | Food |
| Gicquelais 2016 | Influenza vaccine clinic | Universal | Healthcare |
| Pannaraj 2014 | Influenza vaccine clinic | Universal | Healthcare |
| Benjamin-Chung 2020 | Influenza vaccine clinic | Universal | Healthcare |
| Plaspohl 2014 | Influenza vaccine clinic | Universal | Healthcare |
| Ruff 2023 | Mobile dentist (CariedAway) | Universal | Healthcare |
| Darnell 2019 | School nurse | Universal | Healthcare |
| Rodriguez 2013 | School nurse | Universal | Healthcare |
| Leach 2023 | School nurse | Universal | Healthcare |
| Best 2021 | School nurse | Indicated | Healthcare |
| Lim 2023 | School-based health center | Universal | Healthcare |
| Westbrook 2020 | School-based health center | Universal | Healthcare |
| Runton 2016 | School-based health center | Universal | Healthcare |
| Strolin-Goltzman 2014 | School-based health center | Universal | Healthcare |
| Bersamin 2016 | School-based health center | Universal | Healthcare |
| Gruber 2023 | School-based health center | Universal | Healthcare |
| Long 2021 | Telehealth program: school nurses and students virtually visit with a medical provider | Universal | Healthcare |
| Dudovitz 2020 | Vision services: free screening, eye exam, and glasses | Universal | Healthcare |
| Neitzel 2021 | Vision services: free screening, eye exam, and glasses | Universal | Healthcare |
| Hark 2020 | Vision services: free screening, eye exam, and glasses | Universal | Healthcare |
| Glewwe 2018 | Vision services: free screening, eye exam, and glasses | Universal | Healthcare |
| Slavin 2018 | Vision services: free screening, eye exam, and glasses | Universal | Healthcare |
| Gottfried 2017 | School bus system | Universal | Transportation |
| Munoz 2022 | Free public transportation | Universal | Transportation |

# **Supplemental Table 2**. Study characteristics and findings of included studies (n=55).

| **Study** | **Study aims** | **Outcomes** | **Study design** | **Student sample size** | **School sample size** | **Analysis** | **Results** |
| --- | --- | --- | --- | --- | --- | --- | --- |
| Meloche 2020 | Examine the impact of a community school and refugee center on immigrant and refugee youths' outcomes | Attendance; Disciplinary involvement; Academic assessment | Quasi-experiment | 3,426 | 3 | One-Way ANOVA and chi-square | Analyses did not find differences in attendance rates between students in treatment and comparison schools (*p*s>0.05). English Language Learner students in treatment schools had fewer suspensions, and higher GPA, ELA, math, and science grades than those in comparison schools (*p*s< 0.05). 8th grade English Language Learner students in treatment schools had a lower proportion of students with a GPA ≥3.0 (25.7 versus 11.4%), a lower proportion of students failing ELA (12.5 versus 18.4%), a lower proportion of students failing math (6.3 versus 24.0%), and a lower proportion of students with behavioral incidents (18.9 versus 30.0%) than students in comparison schools (*p*s< .05). |
| Biag 2016 | Investigate the association between the community-school programming and attendance and achievement | Attendance; Academic assessment | Descriptive, longitudinal | 5,003 | 6 | Multilevel longitudinal growth modeling | Use of support services was linked to lower attendance rates (B=-0.14, SE=0.07; *p*=0.03) and lower ELA achievement (B=-0.03, SE=0.01, *p*=0.05). Analyses found no association between use of social support services and math achievement (B=-0.02, SE=0.02; *p*=0.14). |
| Pullmann 2013 | Evaluate associations between participation in a school-based family support program and student outcomes | Attendance; Disciplinary involvement; Academic assessment | Descriptive, longitudinal | 25,435 | 65 | Paired t-test, chi-square test, analysis of covariance | Qualitative data from focus groups indicated that school faculty/staff, families, and family support workers believe the program has a positive impact on academic functioning. Quantitative analyses did not find that the program was related to improvements in student attendance, disciplinary actions or standardized test scores. |
| Bates 2019 | Explore how interprofessional team collaboration affects student- outcomes | Attendance; Disciplinary involvement | Descriptive, longitudinal | 241-340 | 4 | Thematic analysis, paired t-tests | Analyses found no difference in absences before versus after CARE in the full sample (*p*=0.65). Among 100 high-risk students, students missed fewer days in the eight weeks following service initiation (M=4.43, SD=3.79) compared to the eight weeks prior (M=5.48, SD=3.22, *p*=0.07), and had fewer referrals in the eight weeks following (M=0.68, SD=1.12), compared to the eight weeks prior (M=1.38, SD=0.99, *p*<0.01). Analyses found no difference in disciplinary referrals before versus after CARE (*p*=0.92). |
| Cuadros-Meñaca 2022a | Examine the effect of BATB on academic achievement | Academic assessment | Quasi-experiment | 97,527 | Not reported | Difference-in-difference | BATB had negative effects on math (ATT=-0.02; 95% CI: -0.04, -0.00), driven by more affluent children (ATT=-0.03; 95% CI: -0.06, -0.01). Analyses found no effects on ELA. ATT by grade indicate positive effects on math for vulnerable children when BATB is adopted earlier (e.g., Grade 4 adoption: ATT=0.10; 95% CI: 0.01, 0.19). |
| Abouk 2022 | Test for the effects of expanding school breakfast access on student achievement | Academic assessment | Quasi-experiment | 6,310 | Not reported | Linear regression | Analyses did not find associations between BATB and reading, math, nor science scores (*p*s>0.05). |
| Cuadros-Meñaca 2023 | Measure the effect of BATB on the likelihood of committing an infraction and the number of infractions | Disciplinary involvement; Physical fighting or bullying | Quasi-experiment | 54,087 | 1,059-1,097 | Difference-in-difference with propensity score matching | Among students with prior misconduct infractions, attending a school with BATB was associated with 0.38 (SE=0.08) fewer misconduct infractions per year. Among all students, attending a school with BATB was associated with 0.02 (SE=0.08) fewer misconduct infractions per year. Analyses found no effects on aggressive behavior (Bs=-0.001; SEs=0.003-0.033) or other infractions (Bs=-0.003-0.005; SEs=0.01-0.14). |
| Anzman-Frasca 2015 | Estimate the impact of BATB on school attendance and academic achievement | Attendance; Academic assessment | Quasi-experiment | Not reported | 446 | Propensity score modeling, generalized linear mixed modeling | BATB was associated with greater overall school attendance rates (95.5% vs 95.3% in the non-BATB group; *p*<0.01). Analyses found no group differences in standardized test performance in math (57.9% vs 57.4% in the non-BATB group; *p*=0.52) or reading (44.9% vs 44.7% in the non-BATB group; *p*=0.70). |
| Gottfried 2022 | Examine whether BATB is linked to better attendance | Attendance | Quasi-experiment | 23,710 | Not reported | Multivariable linear regression with county and child fixed effects | Children in schools with BATB exhibited fewer absences (*p*<0.05) and lower likelihood of chronic absenteeism (*p*<0.05) |
| Hearst 2019 | Examine the impact of BATB on GPA | Academic assessment | Experiment | 636 | 13 | Linear mixed models, latent class analysis | Analyses found no significant intervention effect on GPA postintervention or 1-year follow-up (*p*s>0.05) |
| Cuadros-Meñaca 2022b | Examine whether BATB affects math and literacy scores in third grade | Academic assessment | Quasi-experiment | Not reported | 184 | Synthetic control method | BATB did not impact math or ELA standardized test scores (*p*s>0.05). |
| Kirksey 2021 | Examine the role of BATB in reducing absenteeism | Attendance; Academic assessment | Quasi-experiment | 1,036,384 | 1,883 | Regression discontinuity | Schools with BATB had chronic absence rates that were eight (SE=0.04) to ten (SE=0.05) percentage points lower than schools without BATB (*p*s<0.05). Analyses did not find an effect of BATB on reading or math (*p*s>0.05). |
| Walker 2021 | Assess the effect of BATB on student outcomes | Attendance; Disciplinary involvement | Quasi-experiment | 2906 | 9 | Repeated measures analysis | School absences and school suspension rates were lower for students in the BATB program than in the Cafe program (*p*s<0.05). |
| Corcoran 2016 | Estimate the impact of BATB on students' academic performance and attendance | Attendance; Academic assessment | Quasi-experiment | Not reported | 1,088 | Difference-in-difference | Analyses found no evidence of an impact of BATB on attendance or achievement (in either math or ELA exams; *p*s>0.05). |
| Chand-rasekhar 2023 | Evaluate the impact of BATB on attendance and academic performance | Attendance; Academic assessment | Quasi-experiment | 30,493 | Not reported | Multivariable linear and logistic regression | BATB participants were over 2.5 times more likely to attend school than non-BATB participants (aOR=2.55, 95% CI: 2.23-2.92; *p*<0.001). Analyses found no significant changes in reading (*p*=0.23) and math scores (*p*=0.84). |
| Luan 2022 | Examine the effect of UFB in the classroom on attendance and standardized test performance over 2.5 years compared to UFB served in the cafeteria before school | Attendance; Academic assessment | Experiment | 1,362 | 16 | Weighted generalized estimating equation models | Analyses found no influence of BATB on attendance (B=0.004, SE=0.06; *p*=0.94) or standardized reading exam scores (B=0.02, SE=0.06; *p*=0.79) after 2.5 years. Students in BATB schools had lower standardized math exam scores than those in control schools after 2.5 years, although this difference was small (B=-0.20, SE=0.07; *p*=0.005). |
| Imberman 2014 | Assess the impact of free BATB on students' academic performance and attendance relative to providing free breakfast in the cafeteria | Attendance; Academic assessment | Quasi-experiment | 6,353- 38,425 | 84-87 | Difference-in-difference | Providing free BATB relative to the cafeteria raised math and reading achievement by 0.09 (SE=0.046) and 0.06 (SE=0.034) SD. Analyses did not find that BATB relative to the cafeteria influenced grades (B=-0.010, SE=0.04) or attendance (B=-0.060, SE=0.08). |
| Frisvold 2015 | Investigate the impact of SBP, and a binding state mandate on school participation in an SBP program, on cognitive achievement | Academic assessment | Quasi-experiment | 3,040-53,430 | Not reported | Difference-in-difference, regression discontinuity | A binding state mandate increased math achievement by 0.9-1.8% of the mean, reading by 0.9-2.0% of the mean, and science by 0.8% of the mean. Exceeding the state threshold increased math achievement by 2.6 (SE=1.24) to 7.6 (SE=3.43) points, reading by 4.4 (SE=1.82) to 6.6 (SE=3.01) points, and science by 5.5 (SE=0.39) points. |
| Bartfeld 2019 | Estimate the impact of SBP on school attendance and standardized test scores | Attendance; Academic assessment | Quasi-experiment | 248,328-481,799 | 1,007 | Multivariable linear regression with grade, year, and school fixed effects | Compared with the traditional SBP, UFB was associated with a 0.24 percentage point increase in the percentage of days attended (*p*=0.02) and a 3.5 percentage point decrease in the percentage of students with low attendance (*p*<0.01). UFB was associated with a 0.07 SD increase in math scores (*p*<0.01) and 0.04 SD increase in reading scores (*p*=0.035) among higher-income students. BATB had no associations with attendance in the full sample or subgroups. BATB was associated with lower math scores (0.05 SD, *p*=0.045) for lower-income boys. |
| Ribar 2013 | Investigate student outcomes (attendance rates and standardized test scores) associated with changes in the availability of UFB | Attendance; Academic assessment | Quasi-experiment | 8,078 | 10-22 | Difference-in-difference | Analyses did not find an association between UFB (versus eligibility-based breakfast) and student attendance. At the student level, UFB was associated with a half percent decrease in attendance (SE=0.001; *p*<0.01). Analyses found no evidence that UFB is associated with reading or math test results or with science raw scores. UFB was associated with science test proficiency among economically disadvantaged children (B=0.08; SE=0.03). |
| Bullock 2022 | Examine whether a UFB policy was associated with changes in school attendance | Attendance | Quasi-experiment | Not reported | 150 | General linear mixed models | Analyses did not find increases in SBP participation to be associated with significant changes in attendance (0.003 SE=0.01, *p*=0.9). |
| Leos-Urbel 2013 | Examine the impact of UFB on attendance and academic achievement | Attendance; Academic assessment | Quasi-experiment | 723,843 | 668 | Difference-in-difference | Analyses identified no change in attendance rates (B=0.11-0.14, SE=0.09-0.10), reading scores (B=0.01, SE=0.01), or math scores (B=-0.01-0.02, SE=0.0) after adopting UFB. |
| Williams 2022 | Examine the impact of policy change of UFL on third grade normal curve equivalent reading scores | Academic assessment | Quasi-experiment | Not reported | 574 | One-way ANOVA | Analyses found no differences in third grade reading scores after the policy change to 100% UFL (p>0.05). |
| Gordanier 2020 | Investigate the effect of UFL, through CEP, on academic outcomes of elementary and middle school students | Attendance; Academic assessment | Quasi-experiment | 332,761 | 780 | Difference-in-difference | For elementary school students, CEP led to a 0.061 (SE=0.014) standard deviation increase in math test scores and 0.213 (SE=0.111) fewer absences; analyses found no effect of CEP on reading test scores for elementary students. Analyses did not find any effects of CEP on math test scores, reading test scores, or absences among middle school students. |
| Schwartz 2020 | Estimate the impact of UFM on student outcomes, including performance on test scores and attendance | Attendance; Academic assessment | Quasi-experiment | 645,204 | 233-1103 | Difference-in-difference | UFM increased math and ELA scores for middle school students by 0.036 (SE = 0.014) and 0.030 (SE = 0.011) standard deviations, respectively. Analyses found no effects on attendance. |
| Taylor 2020 | Analyze the impacts of UFM on student academics and behavior | School engagement; Academic assessment | Cross-sectional | Not reported | 57 | Qualitative coding | Eighty-three percent of school staff agreed or strongly agreed that UFM make students more ready to learn; 64.4% agreed that students' academic performance had improved with universal meals. Most respondents (59.1%) agreed that student behavior improved. Most school nurse participants disagreed that student issues had declined (55%). |
| Andre-yeva 2021 | Assess the impacts of CEP on attendance and academic achievement among low-income communities | Attendance; Academic assessment | Quasi-experiment | 17,800 | 3,200 | Difference-in-difference | UFM improved daily school attendance by 0.24 percentage points (*p*< 0.01). Models for academic achievement outcomes did not pass the parallel trend assumption in the full analytic sample. Among Hispanic children, there was a CEP-attributable increase in reading scores (0.06 SD, *p*<0.1). |
| Ruffini 2022 | Evaluate the effect of CEP on academic performance | Academic assessment | Quasi-experiment | Not reported | Not reported | Difference-in-difference | Analyses did not find that CEP improved reading or math performance overall. Among exposed districts with the largest gains in access to free meals, CEP improved overall math performance by 0.02 SD (SE=0.009). |
| Gordon 2021 | Examine whether schoolwide free meals affect disciplinary outcomes, focusing on the use of suspensions | Disciplinary involvement | Quasi-experiment | 8,594,838-22,423,824 | 14,178-47,767 | Difference-in-difference | Schoolwide free meals reduced suspensions by approximately one percentage point (SE=0.46; *p*<0.05) for white male elementary students; analyses did not find associations overall for elementary students, middle/high school students, or for other subgroups. |
| Bartfeld 2020 | Estimate the association between CEP and school attendance among elementary school students | Attendance | Quasi-experiment | Not reported | 145 | Difference-in-difference | Implementing the CEP had no association with attendance in the initial year. The second year of CEP was associated with a 3.5 percentage point reduction in the percentage of students with low attendance (*p*=0.045), driven by economically disadvantaged students. |
| Kurtz 2020 | Examine the effects of BackPack on end of grade test scores and absenteeism | Attendance; Academic assessment | Quasi-experiment | 32,690-36,986 | 82 | Difference-in-difference-in-difference | Having a BackPack program increased reading test scores by 0.093 (SE=0.039) standard deviations (*p*<0.05). Analyses found no effects of the BackPack program on math test scores (B=0.07, SE=0.06; *p*>0.05) or absences (B=0.146, SE=0.152; *p*>0.05). |
| Fiese 2020 | Determine the potential of the Backpack program to affect school attendance | Attendance | Cross-sectional | 444 | 16 | Chi-square | There were fewer absences on Fridays for students who participated in the BackPack program compared to Mondays-Thursdays; this was not true for non-recipients (p<0.05). |
| Gicquelais 2016 | Quantify the association between student vaccination levels and student absenteeism during influenza season | Attendance | Cross-sectional | Not reported | 422 | Linear regression | A one-percent increase in student vaccination rates was associated with 0.027% (adjusted; unadjusted: 0.031%) fewer days absent (*p*<0.01). |
| Pannaraj 2014 | Evaluate the impact of a school vaccine clinic on absenteeism | Attendance | Quasi-experiment | 4,455 | 8 | Multiple logistic regression | All-cause absentee rates among students were higher in comparison schools compared with intervention schools (4.2 vs 3.9 days per 100 school days; *p*=0.02). Fewer children in comparison schools had zero days of absenteeism compared with those in intervention schools (549 [26.3%] vs. 793 [33.5%]; *p*<0.01) |
| Benjamin-Chung 2020 | Assess whether the school vaccine clinic was associated with reduced school absences | Attendance | Quasi-experiment | Not reported | 84 | Difference-in-difference | In 2016-2017 and 2017-2018, the illness-specific absence rate per 100 days was lower in the intervention district compared to the comparison district (2016-2017 DID: -0.63 [95% CI: -1.14, -0.13], p=0.014]; 2017-2018 DID: -0.80 [95% CI: -1.28, -0.31], *p*=0.001]). For all-cause absences, the DID estimates during influenza season were not statistically significant in any years of the program. |
| Plaspohl 2014 | Assess the associations between a school vaccine program and student absences for elementary school students | Attendance | Quasi-experiment | 4,674-5,201 | 8 | Independent t-tests | Students in the two years pre-program were absent for 0.36 fewer days compared to students in the two program years (*p*<0.0001; 95% CI=0.28, 0.44). Vaccinated students in the two program years were absent for 0.64 fewer days as compared to unvaccinated students in the two program years (*p*<0.0001, 95% CI=0.51, 0.78). |
| Ruff 2023 | Examine the effect of school-based dental programs on student attendance | Attendance | Experiment | Not reported | 52 | Mixed-effects linear regression, 2-limit tobit regression | Schools recorded ~944 fewer absences during treatment compared to nontreatment years (95% CI=–1,739 to –149). Analyses found no difference in absences between treatment and comparison schools (*p*>0.05). |
| Darnell 2019 | Examine the relationship between school nurse presence and graduation rates, absenteeism, and ACT scores | Attendance; Graduation; Academic assessment | Cross-sectional | Not reported | 230 | Multivariable linear regression | Schools with a full-time nurse had a lower absentee rate (6.3% versus 6.98%; B=-0.76, p=0.01), higher student graduation rate (83.1% versus 76.9%; B=5.67, *p*<0.001), and higher ACT scores (18.9 versus 18.5 ACT; B=0.51, *p*=0.04) than schools with no nurse. |
| Rodriguez 2013 | Examine the change in school absenteeism associated with full-time nurses | Attendance | Quasi-experiment | 583-6,081 | 9 | Logistic regression analysis | Students in demonstration schools had fewer absences due to illness (3.03 days) compared to students in comparison schools (3.51 days; *p*<0.05). |
| Leach 2023 | Evaluate the effects of an existing LPN-based school nurse program on attendance and chronic absenteeism | Attendance | Quasi-experiment | 16,025 | 46 | Multi-level random intercepts regression models, with matched comparison schools | Analyses found no associations between being in schools with a nurse and chronic absenteeism or attendance (*p*s>0.05). |
| Best 2021 | Examine the impact of student-to-nurse ratios on education outcomes of students receiving services for asthma and diabetes | Attendance; Academic assessment | Descriptive, longitudinal | 1.43 million | Not reported | Generalized linear modeling | Lower student to school nurse ratios were associated with fewer absences and improved grades (*p*<0.05). |
| Lim 2023 | Examine if attendance improves after students receive care at an SBHC | Attendance | Quasi-experiment | 244,076 | 17 | Propensity weighted linear regression models | An SBHC visit was associated with a 0.65 percentage-point increase in school days attended per month (*p*<0.01). An SBHC mental health visit was associated with a 2.33 percentage-point increase in school days attended per month (*p*<0.001). |
| West-brook 2020 | Examine the association between SBHC opening and high school graduation rates | Graduation | Quasi-experiment | Not reported | 132 | Panel fixed effects models | Opening an SBHC during the study period was associated with a 4.1 percentage point increase in the overall graduation rate (*p*=0.08). |
| Runton 2016 | Investigate which health risk behaviors were affected by a new SBHC | Physical fighting or bullying; Substance use | Descriptive, longitudinal | 750-1,169 | Not reported | Logistic regression | Analyses did not find associations between SBHCs and the likelihood of a student carrying a weapon, being in a physical fight, or smoking a cigarette (*p*s>0.05). |
| Strolin-Goltzman 2014 | Evaluated associations between SBHC use and academic performance | Attendance; Academic assessment; Grade repeat | Cross-sectional | 793 | 3 | Structural equation modeling | SBHC users in the middle and high schools had GPAs that were 2.5 points higher than those for nonusers (*p*<0.05). 90 percent of middle and high school SBHC users advanced to the next grade, compared to 83 percent of non-users (*p*<0.05). SBHC users were late on an average of 9.5 days, whereas nonusers averaged five tardy days (*p*<0.05). There was no difference in attendance between users and nonusers (*p*>0.05). |
| Bersamin 2016 | Examine the association of SBHC with academic achievement and college preparation | Graduation | Quasi-experiment | Not reported | 296-810 | Regression with propensity score matching | SBHC was positively associated with college preparation: ACT (B=4.49, *p*<0.01), AP (B=5.65, *p*<0.01), and SAT (B=7.35, *p*<0.01) participation rates. Analyses found no significant association between SBHC presence and graduation rates nor meeting state university graduation requirements. |
| Gruber 2023 | Examine the effects of SBHC use on attendance among high school students | Attendance | Descriptive, longitudinal | 413 | 6 | Autoregressive cross-lagged structural equation models | Analyses did not find that SBHC use directly (B<0.01, SE<0.01) nor indirectly (B<0.01, SE<0.01) predicted absenteeism through its effect on health or physical activity. |
| Long 2021 | Evaluate the effectiveness of telehealth at reducing absences compared to standard school-based wellness services | Attendance | Quasi-experiment | 4,203 | 6 | Negative binomial regression model | Absences were 7-8% higher in students not enrolled in telehealth in 2015-2016 (7.20 vs. 6.64 days) and 2016-2017 (7.81 vs. 7.32). In the adjusted negative binomial regression model, students enrolled in the telehealth program were absent 7.7% (p=0.025; 95% CI: 1.0%, 14%) fewer days per year than non-telehealth students. |
| Dudovitz 2020 | Test whether participation in a free school-based vision-care program was associated with improved school performance for low-income minority students in Los Angeles | Academic assessment | Quasi-experiment | 23,799 | 24 | Multilevel linear regressions | Results revealed no significant change in math achievement rank or work habits rank over the two years after receiving glasses. Students demonstrated improved ELA achievement rank by 4.5 percentile points in the second year after receiving glasses (*p*=0.02). |
| Neitzel 2021 | Assess the effect of a school-based vision program on academic achievement among students in grades 3-7 | Academic assessment | Experiment | 2,304 | 120 | Hierarchical linear modeling | The vision program was associated with higher reading score (ES=0.09; *p*=0.02) and math scores among elementary students (ES=0.03; *p*<0.001) during school year 2016-2017. The intervention did not show a sustained impact at 2 years for college or career testing. |
| Hark 2020 | Assess the impact of eyeglass administration after a vision-screening program on standardized testing scores in school-aged children | Academic assessment | Quasi-experiment | 349-4,523 | Not reported | Cumulative link mixed regression | Initially high reading performances were less likely to decline among students who received glasses compared to children who either passed their vision screen (with or without glasses they had) or children who failed their screen and did not receive glasses (OR=4.36, *p*<0.001). Analyses did not find an effect of the intervention for children with initially moderate or low reading performances. |
| Glewwe 2018 | Quantify the impact of vision screening, accompanied by eye exams and eyeglasses on student outcomes, including test scores, attendance rates, and discipline incidents. | Attendance; Disciplinary involvement; Academic assessment | Experiment | 14,572-15,422 | 76 | Regression modeling | Students in full-treatment schools were 2.0 percentage points more likely to be proficient in reading than their peers in control schools (*p*<0.01). Analyses did not find differences for math scores, the total number of absences, unexcused absences, nor the number of behavioral referrals or suspensions between students in the full-treatment schools and control schools. |
| Slavin 2018 | Assess the effect of providing eyeglasses on the reading performance of disadvantaged students | Academic assessment | Quasi-experiment | 262-317 | 12 | Analysis of covariance (ANCOVA) | Students who received glasses improved more on reading scores than those who never needed glasses (ES=0.16, *p*<0.05). |
| Gottfried 2017 | Identify if kindergartners who took the bus had fewer absences and lower risk of being chronically absent compared to those who went to school by other means | Attendance | Cross-sectional | 11,100-14,370 | Not reported | Multivariable linear regression and multivariable linear probability models | Children who took the bus to school had fewer absent days (B=-0.28, SE=0.10) and were less likely to be chronically absent (B=-0.02, SE=0.01) than students who travelled to school via other means. |
| Munoz 2022 | Estimate the effect of free bus fare on the school attendance behavior of middle and high school students | Attendance | Quasi-experiment | Not reported | 734 | Synthetic control with difference in difference estimator | The free public transportation program was associated with a decrease of around 1 percentage point in school attendance and an increase of 2-4 percentage points in chronic absenteeism. |

Notes: Abbreviations: ACT (American College Test); ATT (average treatment effect on the treated); AP (Advanced Placement); BATB (breakfast after the bell); CEP (Community Eligibility Provision); CI (confidence interval); DID (difference-in-difference); ELA (English Language Arts); ES (effect size); GPA (grade point average); OR (odds ratio); RCT (randomized controlled trial); SBHC (school-based health center); SBP (School Breakfast Program); SD (standard deviation); SE (standard error); UFB (universal free breakfast); UFL (universal free lunch); UFM (universal free meals)

# **Supplemental Table 3**. Settings and populations of included studies (n=55).

| **Study** | **Location** | **Study years** | **School level** | **Grade range** | **Age range** | **Student gender** | **Student race** |
| --- | --- | --- | --- | --- | --- | --- | --- |
| Meloche 2020 | Sansom Valley School District, Pennsylvania | 2014 - 2016 | Middle school | 6-8 | Not reported | Female: 48.2% Male: 51.8% | African American: 20.3% Asian (not Hispanic): 6.6% Multiracial (not Hispanic): 3.1% White: 15.4%  Hispanic: 54.6% |
| Biag 2016 | Redwood City School District - Bay Area, California | 2006 - 2013 | Elementary school; Middle school; Elementary-middle school mixed | K-8 | Not reported | Male: 51.7% Female: 48.3% | Caucasian: 2.9%  Latino: 91.6% Other ethnicity: 4% |
| Pullmann 2013 | Seattle, Washington | 2009 - 2011 | Elementary school | K-5 | 4-13 | Male: 50.7% Female: 49.3% | White: 44.7% Asian: 16.9% Native American: 1% Black: 17.9% Multiracial: 6.2% Native Hawaiian/Pacific Islander: 0.5%  Hispanic: 12.9% |
| Bates 2019 | Large school district in the Intermountain West | 2015 - 2016 | Elementary school | pre-K-5 | Not reported | Male: >65% | African American: 5% White or Caucasian: 49% Native American: 1% Pacific Islander: 1% Multiracial: 5%  Hispanic: 39% |
| Cuadros-Meñaca 2022a | Arkansas | 2008 - 2019 | Elementary school; Middle school | 4-8 | Not reported | Boys: 52.5% | Minority: 26.5% |
| Abouk 2022 | Dallas, Texas; West Virginia; New Mexico; Nevada; Colorado | 2011 - 2016 | Elementary school | 1-5 | Not reported | Male: 51.1% | White non-Hispanic: 53.6% Black non-Hispanic: 12.3% Other races: 10.1% Hispanic: 23.9% |
| Cuadros-Meñaca 2023 | Arkansas | 2008 - 2019 | Elementary school; Middle school | K-7 | Not reported | Not reported | Not reported |
| Anzman-Frasca 2015 | Large urban school district | 2012 - 2013 | Elementary school | K-6 | Not reported | Not reported | Black: 9.4% White: 11.4% Asian: 4.2% AIAN: 0.4% Pacific Islander: 0.5% Latino: 72.2%  Filipino: 1.7% |
| Gottfried 2022 | United States | 2010 - 2012 | Elementary school | K-1 | Not reported | Male: 51.5% | Asian: 9.3% Black: 17.7% Other race: 8%  Hispanic: 26.9% |
| Hearst 2019 | Rural Minnesota | 2013 - 2016 | High school | 9-10 | Average = 15.2 | Female: 54% Male: 46% | White: 76% Non-White: 24% |
| Cuadros-Meñaca 2022b | Arkansas | 2008 - 2019 | Elementary school | 3rd grade | Not reported | Boys: 51.1% | Non-White: 29.7% |
| Kirksey 2021 | Colorado; Nevada | 2013 - 2016 | Elementary school; Middle school; High school | Not reported | Not reported | Not reported | Asian: 3% Black: 5%  Latinx: 35% |
| Walker 2021 | Large, urban district in the Northeastern United States | 2013 - 2016 | Elementary school; Middle school; Elementary-middle school mixed | K-8 | Not reported | Not reported | Not reported |
| Corcoran 2016 | New York, New York | 2001 - 2012 | Elementary school; Middle school | K-8 | Not reported | Male: 50.7% | Asian: 11.4% Black: 34.5% White: 13.4%  Hispanic: 39.9% |
| Chandrasekhar 2023 | Dallas Independent School District - Dallas, TX and Dallas suburbs | 2017 - 2019 | Elementary school; Middle school; High school | 5-12 | 9-19 | Male: 50.7%  Female: 49.3% | Non-Hispanic Black: 20.5% Non-Hispanic White: 4.6% Asian: 1.2% AIAN: 0.2% Native Hawaiian/Pacific Islander: 0.1% Multiracial: 0.7% Missing: 0.03%  Hispanic/Latino: 72.8% |
| Luan 2022 | Philadelphia, Pennsylvania | 2013 - 2016 | Elementary school; Middle school; Elementary-middle school mixed | 4-6 | Not reported | Male: 48.6% Female: 51.4% | Asian: 6.1% Black: 66.6% White: 7.3% Multiracial or other: 2.9%  Hispanic: 17.1% |
| Imberman 2014 | Large urban school district in the Southwest US | 2002 - 2010 | Elementary school | 1-5 | Not reported | Female: 48% | Black: 23% White: 1.5%  Hispanic: 73.5% |
| Frisvold 2015 | United States | 2003 - 2004 | Elementary school | 4-5 | Not reported | Female: 49.1-49.2% | Black: 14-24.3% Other race/ethnicity: 3.9-8.6% White: 60.2-62% Hispanic 11.6%-15.4% |
| Bartfeld 2019 | Wisconson, excluding Milwaukee | 2009 - 2014 | Elementary school | Grades 1-5 | Not reported | Male: 51.4%  Female: 48.6% | White: 78.5%  Black: 6%  Asian: 4.2% Native American: 1.7%  Hispanic: 9.5% |
| Ribar 2013 | Guilford County, North Carolina | 2007 - 2009 | Elementary school | 1-5 | Not reported | Female: 48.9% | Black: 65.9%  Hispanic: 17.1% |
| Bullock 2022 | Large urban school district in the Southeast United States | 2006 - 2015 | Elementary school; Middle school; High school; Elementary-middle school mixed; Middle-high school mixed; Elementary-middle-high school | K-12 | Not reported | Not reported | African American: 37%  White: 25%  Asian American: 7%  Multiracial: 3%  Native American/Pacific Islander: <1%  Hispanic: 28% |
| Leos-Urbel 2013 | New York, New York | 2002-2004 | Elementary school; Middle school | 3-8 | Not reported | Not reported | Black: 32.3% Asian: 14.2% White: 17.8%  Hispanic: 35.7% |
| Williams 2022 | Tennessee | 2013 - 2014 | Elementary school | 3rd grade | Not reported | Not reported | Not reported |
| Gordanier 2020 | South Carolina | 2014 - 2016 | Elementary school; Middle school | 3-8 | Not reported | Female: 48.8% | White: 53.5% Black: 35.4% Asian and Pacific Islander: 2.1% Native Indian: 0.6%  Hispanic: 8.4% |
| Schwartz 2020 | New York, New York | 2013 | Middle school | 6-8 | Not reported | Female: 50.3% | White: 15% Asian: 17.4% Black: 28.1%  Hispanic: 39.5% |
| Taylor 2020 | Vermont | 2016 - 2017 | Elementary school; Middle school; High school | K-12 | Not reported | Not reported | Not reported |
| Andreyeva 2021 | 41 states; urban, suburban, and rural areas | 2010 - 2016 | Elementary school | K-5 | Not Reported | Male: 51.3% Female: 48.7% | Non-Hispanic White: 47.6% Non-Hispanic Black: 11.3%  Asian: 8.8%  Other race: 5.8%  Hispanic: 26.4% |
| Ruffini 2022 | United States | 2006 - 2018 | Elementary school; Middle school | 3-8 | Not reported | Not reported | Black: 22.4%  Hispanic: 16.7% |
| Gordon 2021 | United States | 2012 - 2017 | Elementary school; Middle school; High school | Not reported | Not reported | Not reported | Black: 12.3-13.9% White: 46.5-60.4%  Hispanic: 18.6-20.5% |
| Bartfeld 2020 | Wisconsin, excluding Milkwaukee | 2013 - 2016 | Elementary school | 1-5 | Not reported | Not reported | Non-white: 48.9% |
| Kurtz 2020 | 12 counties in Northwestern North Carolina | 2007-2013 | Elementary school | 3-5 | Average: 10.37 | Female: 49.2% | White: 27.6% |
| Fiese 2020 | East Central Illinois | 2011 - 2012 | Elementary school | K-5 | Not reported | Boys: 51% | African American: 31.7% Caucasian: 54.4% Multiracial: 6.6%  Latino: 6.6% |
| Gicquelais 2016 | Arkansas | 2012 - 2013 | Elementary school; Middle school; High school; Middle-high school mixed | K-12 | Not reported | Not reported | Not reported |
| Pannaraj 2014 | Los Angeles County, California | 2010 - 2011 | Elementary school | K-6 | 5-13 | Male: 52.1% | Asian: 26.7% White: 2.0% Black: 0.6% Other: 4.3%  Hispanic: 66.3% |
| Benjamin-Chung 2020 | Oakland Unified School District, California | 2011 - 2018 | Elementary school | K-5 | Not reported | Not reported | White: 43.8% Black or African American: 22.4% Asian: 17.2% Other Race: 8.6% Native Hawaiian/Pacific Islander: 0.6% Multiracial: 6%  Hispanic or Latino: 28.8% |
| Plaspohl 2014 | Effingham County, Georgia | 2007-2012 | Elementary school | K-5 | Not reported | Male: 52% Female: 48% | White non-Hispanic: 77% Black: 14% Multiracial: 4% Asian/Pacific Islander: 1% AIAN/Unknown: <1%  Hispanic: 4% |
| Ruff 2023 | New York, New York | 2016 - 2021 | Unknown | Not reported | Not reported | Not reported | Not reported |
| Darnell 2019 | Kentucky | 2013 - 2014 | High school | 9-12 | Not reported | Not reported | Not reported |
| Rodriguez 2013 | San Jose Unified School District, California | 2006 - 2009 | Elementary school; Middle school | Pre-K-8 | Not reported | Not reported | Hispanic/Latino: 77.4% |
| Leach 2023 | Jefferson County, Kentucky | 2017-2019 | Elementary school | 1-5 | Not reported | Female: 48.5% Male: 51.5% | Black: 45.5% Other Race/Ethnicity: 9.4% White: 30%  Hispanic/Latinx: 15.5% |
| Best 2021 | All school districts in North Carolina | 2011 - 2016 | Elementary school; Middle school; High school | K-12 | Not reported | Not reported | Not reported |
| Lim 2023 | Southern California | 2015 - 2020 | Unknown | Not reported | Not reported | Female: 56.6% Male: 43.4% | Asian: 5.9% Black: 6.0% White: 10.5% Multiracial: 0.2% Unknown: 0.7%  Latino: 76.6% |
| Westbrook 2020 | Colorado | 2000 - 2018 | High school | 9-12 | Not reported | Women: 48.9% | Minority: 22.8% |
| Runton 2016 | Virginia; North Carolina | 2007 - 2011 | Middle school; High school | 7, 11-12 | 11 to >18 | Female: 49.8-54.9% Male: 45.1-50.2% | White: 25.6-25.9% Black: 38.6-41.7% Other/multiple: 11.2-11.7%  Hispanic/Latino: 21-24.3% |
| Strolin-Goltzman 2014 | Large, northeastern urban metropolis | Not reported | Elementary school; Middle school; High school | Not reported | Not reported | Female: 48.5% Male: 51.3% | African: 2.5% Black or African American: 19.6% Asian/Pacific Islander: 5.2% Non-Hispanic White: 6.6% Native American: 1.1% Multiracial: 4.6%  Hispanic: 60.2% |
| Bersamin 2016 | California | 2012 | High school | 9-12 | Not reported | Not reported | Not reported |
| Gruber 2023 | Michigan | 2006 - 2009 | High school | Not reported | Average: 14.4 (SD 0.63)-16.6 (SD 0.62) years | Male: 45-45.4% Female: 54.6-55% | Black: 33.1-38% White: 38-43.6% Not Black or White: 22.5-24.9% |
| Long 2021 | Maryland | 2014 - 2017 | Elementary school | PreK-5 | Not reported | Male: 52.2% Female: 47.8% | Non-Hispanic White: 24.3% Non-Hispanic Black: 45.1% Other Race/Ethnicity: 10.7%  Hispanic: 19.7% |
| Dudovitz 2020 | Los Angeles, California | 2013 - 2016 | Elementary school | 1-5 | Not reported | Male: 45.6% | African American: 18%  Latino: 76% |
| Neitzel 2021 | Baltimore, Maryland | 2015 - 2019 | Elementary school; Middle school | 3-7 | Not reported | Female: 54.7% | Black: 77.6% White: 18.8% Asian: 1.4%  Latinx: 16.8% |
| Hark 2020 | The School District of Philadelphia, Pennsylvania | 2014 - 2015 | Elementary school | K-5 | 6-13 | Girls: 48.7% Boys: 50.7% Unknown: 0.6% | Asian: 30.7% African American: 29.2% White: 18.9% Multiracial/other: 9.8%  Hispanic: 11.5% |
| Glewwe 2018 | Central Florida | 2010 - 2013 | Elementary school | 4-5 | Not reported | Female: 49% | White: 26.4% Asian: 2.2% Black: 28.2% Multiracial: 3.4%  Hispanic: 35.8% |
| Slavin 2018 | Baltimore, Maryland | 2014 - 2015 | Elementary school | 2-3 | Not reported | Not reported | African American: 84.5% White: 4.4%  Hispanic: 8.5% |
| Gottfried 2017 | United States | 2010 - 2011 | Elementary school | Kindergarten | Not reported | Male: 51.5% | Black: 13.7% Asian: 3.8% Other: 5.2% |
| Munoz 2022 | Leon County, Florida | 2003-2019 | Middle school; High school | 6-12 | Not reported | Not reported | African American: 43.6%  White: 42.1%  Hispanic: 6% |

Abbreviations: AIAN (American Indian and Alaska Native)

# **Supplemental Table 4**. Quality assessment for qualitative studies (n=1).

| **Study** | **Is the qualitative approach appropriate to answer the research question?** | **Are the qualitative data collection methods adequate to address the research question?** | **Are the findings adequately derived from the data?** | **Is the interpretation of results sufficiently substantiated by data?** | **Is there coherence between qualitative data sources, collection, analysis and interpretation?** | **Overall rating** |
| --- | --- | --- | --- | --- | --- | --- |
| Taylor 2020 | Yes | No | No | No | No | Low |

# **Supplemental Table 5**. Quality assessment for randomized controlled trials (n=5).

| **Study** | **Is randomization appropriately performed?** | **Are the groups comparable at baseline?** | **Are there complete outcome data?** | **Are outcome assessors blinded to the intervention provided?** | **Did the participants adhere to the assigned intervention?** | **Overall rating** |
| --- | --- | --- | --- | --- | --- | --- |
| Glewwe 2018 | Yes | Yes | No | Can't tell | Can't tell | High |
| Hearst 2019 | Can't tell | Can't tell | Yes | Can't tell | Yes | Moderate |
| Luan 2022 | Yes | Yes | Can't tell | Can't tell | Can't tell | Moderate |
| Neitzel 2021 | Yes | Yes | No | Can't tell | Can't tell | Moderate |
| Ruff 2023 | Yes | Can't tell | Yes | Can't tell | Yes | Moderate |

# **Supplemental Table 6**. Quality assessment for non-randomized quantitative studies (n=49).

| **Study** | **Are the participants representative of the target population?** | **Are measurements appropriate regarding both the outcome and intervention (or exposure)?** | **Are there complete outcome data?** | **Are the confounders accounted for in the design and analysis?** | **During the study period, is the intervention administered (or exposure occurred) as intended?** | **Overall rating** |
| --- | --- | --- | --- | --- | --- | --- |
| Abouk 2022 | Yes | Yes | Yes | No | Can't tell | Moderate |
| Andreyeva 2021 | Yes | Can't tell | Yes | No | Can't tell | Moderate |
| Anzman-Frasca 2015 | Yes | Yes | Yes | Yes | Yes | High |
| Bartfeld 2019 | Yes | Yes | Yes | Yes | Yes | High |
| Bartfeld 2020 | Yes | Yes | Yes | Yes | Yes | High |
| Bates 2019 | Can't tell | Can't tell | Yes | No | Can't tell | Low |
| Benjamin-Chung 2020 | Yes | No | Yes | Yes | Can't tell | Moderate |
| Bersamin 2016 | Yes | Yes | Yes | Yes | Can't tell | High |
| Best 2021 | Yes | Can't tell | Yes | No | Yes | Low |
| Biag 2016 | Yes | Yes | Yes | No | Yes | Moderate |
| Bullock 2022 | Yes | Yes | Yes | No | Yes | Moderate |
| Chandrasekhar 2023 | Yes | Yes | Yes | No | No | Moderate |
| Corcoran 2016 | Yes | Yes | Yes | Yes | Yes | High |
| Cuadros-Meñaca 2022a | Yes | Can't tell | Yes | Yes | Yes | High |
| Cuadros-Meñaca 2022b | Yes | Yes | Yes | Yes | Yes | High |
| Cuadros-Meñaca 2023 | No | No | Yes | Can't tell | Yes | Low |
| Darnell 2019 | Yes | Yes | Yes | No | Yes | Moderate |
| Dudovitz 2020 | Yes | Yes | Yes | No | Yes | Moderate |
| Fiese 2020 | Can't tell | Can't tell | No | No | Can't tell | Low |
| Frisvold 2015 | Yes | Yes | Can't tell | Yes | Can't tell | Moderate |
| Gicquelais 2016 | Yes | Yes | Yes | No | Yes | Moderate |
| Gordanier 2020 | Yes | Yes | Yes | Yes | Yes | High |
| Gordon 2021 | Yes | No | Yes | No | Can't tell | Moderate |
| Gottfried 2017 | Yes | Yes | Yes | No | Can't tell | Moderate |
| Gottfried 2022 | Yes | Yes | Yes | Yes | Yes | High |
| Gruber 2023 | Can't tell | Yes | No | No | Yes | Low |
| Hark 2020 | Yes | Yes | Yes | No | Yes | Moderate |
| Imberman 2014 | Yes | Yes | Can't tell | Yes | Yes | High |
| Kirksey 2021 | Yes | Yes | Yes | Yes | Yes | High |
| Kurtz 2020 | Yes | Yes | Yes | Yes | Can't tell | Moderate |
| Leach 2023 | Yes | Yes | Yes | No | Can't tell | Moderate |
| Leos-Urbel 2013 | Yes | Yes | Can't tell | Yes | Yes | High |
| Lim 2023 | Yes | Yes | Yes | No | Can't tell | Moderate |
| Long 2021 | Yes | Yes | Yes | No | Yes | Moderate |
| Meloche 2020 | Yes | Yes | Can't tell | No | Can't tell | Low |
| Munoz 2022 | Yes | Yes | Yes | Yes | Can’t tell | High |
| Pannaraj 2014 | Yes | Yes | Can't tell | No | Yes | Moderate |
| Plaspohl 2014 | Yes | Yes | Yes | No | Yes | Moderate |
| Pullmann 2013 | Yes | Yes | Yes | No | Yes | Moderate |
| Ribar 2013 | Yes | Yes | Yes | Yes | Yes | High |
| Rodriguez 2013 | Yes | No | Yes | Yes | Yes | Moderate |
| Ruffini 2022 | Yes | Yes | Can't tell | Yes | Yes | High |
| Runton 2016 | Can't tell | Yes | Yes | No | Can't tell | Low |
| Schwartz 2020 | Yes | Yes | Yes | Yes | Yes | High |
| Slavin 2018 | Yes | Yes | Yes | No | Yes | Moderate |
| Strolin-Goltzman 2014 | Yes | Yes | Can't tell | No | Yes | Moderate |
| Walker 2021 | Yes | Yes | Yes | No | Can't tell | Moderate |
| Westbrook 2020 | Yes | Yes | Yes | No | Can't tell | Low |
| Williams 2022 | Yes | Yes | Yes | No | Yes | Low |

# **Supplemental Results 1.** Evidence quality.

Although the Mixed Methods Appraisal Tool (MMAT) does not provide an overall quality score, we used the methodology-specific screening questions to classify studies as high, moderate, or low quality. We also identified common strengths and limitations within these study combinations.

Studies that evaluated the effects of interventions providing food on school performance were largely of moderate to high quality. Most studies were quasi-experimental (n=18), with few experiments (n=2) and one cross-sectional analysis. The main issue limiting study quality was inadequate covariates or study design to control for potential confounders. Yet, many studies worked to address this limitation by incorporating time series data and quantitative methods to obtain comparable untreated schools/districts.

Studies that evaluated the effects of interventions providing food on school participation were largely of moderate to high quality and quasi-experimental (n=16), with two cross-sectional analyses and one experimental. Strengths and limitations were similar to studies focused on the effects of providing food on school performance. Of note, studies operationalized attendance in different ways: attendance rate (the proportion of school days that a student attends), number of absences, and chronically absent (an indicator such as missing 10% or more school days). We did not, however, find the pattern of results to change between these operationalizations.

Studies that evaluated the effects of interventions delivering healthcare on school participation were largely of moderate quality, with half of the studies being quasi-experimental (n=7), several being descriptive (n=5), and two studies being experimental. Common limitations included inadequate control for potential confounding and unclear intervention fidelity.

Studies that evaluated the effects of interventions delivering healthcare on school performance were largely of moderate quality, with half of the studies being quasi-experimental (n=5), several being descriptive (n=3), and two studies being experimental. As with evaluations for school participation outcomes, common limitations included inadequate control for potential confounding and unclear intervention fidelity.

Overall, studies that focused on interventions to connect families to resources were of moderate to low quality, with most being descriptive (n=3) and only one being quasi-experimental. Common study limitations, regardless of outcome, included potential confounding, vague intervention descriptions, and unclear intervention fidelity.

Overall, studies that focused on the effect of interventions providing food on school safety were of moderate to low quality, with most being quasi-experimental (n=3) and one being a cross-sectional analysis. The main limitation of these studies included inadequate covariates or study design to control for potential confounders. Given known biases in student suspension and expulsion data, however, these studies also experienced additional limitations in measuring outcomes.

The two studies that evaluated the effects of interventions providing healthcare on school safety, one experimental and one descriptive analysis, were of mixed quality. The limitation of these studies included inadequate covariates or study design to control for potential confounders. Given known biases in student suspension and expulsion data, however, these studies also experienced additional limitations in measuring outcomes.

These studies, one experimental and one descriptive analysis, were of mixed quality. The main limitation of these studies included inadequate covariates or study design to control for potential confounders. Given known biases in student suspension and expulsion data,^1,2^ however, these studies also experienced additional limitations in measuring outcomes.

Studies evaluating the effect providing transportation on school attendance, one cross-sectional and one quasi-experimental were of moderate to high quality. The main limitation of these studies included inadequate outcome measures or study design to control for potential implementation confounders.

**References**

1.	Erickson JH, Pearson J. Excluding whom? Race, gender, and suspension in high school. *Educ Urban Soc*. 2022;54(4):389-422.

2.	Owens J, McLanahan SS. Unpacking the drivers of racial disparities in school suspension and expulsion. *Soc Forces*. 2020;98(4):1548-1577.
